# Rab39a Interacts with Phosphatidylinositol 3-Kinase and Negatively Regulates Autophagy Induced by Lipopolysaccharide Stimulation in Macrophages

**DOI:** 10.1371/journal.pone.0083324

**Published:** 2013-12-13

**Authors:** Shintaro Seto, Keiko Sugaya, Kunio Tsujimura, Toshi Nagata, Toshinobu Horii, Yukio Koide

**Affiliations:** 1 Department of Infectious Diseases, Hamamatsu University School of Medicine, Hamamatsu, Shizuoka, Japan; 2 Department of Health Science, Hamamatsu University School of Medicine, Hamamatsu, Shizuoka, Japan; 3 Executive Director, Hamamatsu University School of Medicine, Hamamatsu Hamamatsu, Shizuoka, Japan; Universidad de Costa Rica, Costa Rica

## Abstract

Rab39a has pleiotropic functions in phagosome maturation, inflammatory activation and neuritogenesis. Here, we characterized Rab39a function in membrane trafficking of phagocytosis and autophagy induction in macrophages. Rab39a localized to the periphery of LAMP2-positive vesicles and showed the similar kinetics on the phagosome to that of LAMP1. The depletion of Rab39a did not influence the localization of LAMP2 to the phagosome, but it augments the autophagosome formation and LC3 processing by lipopolysaccharide (LPS) stimulation. The augmentation of autophagosome formation in Rab39a-knockdown macrophages was suppressed by Atg5 depletion or an inhibitor for phosphatidylinostol 3-kinase (PI3K). Immunoprecipitation analysis revealed that Rab39a interacts with PI3K and that the amino acid residues from 34^th^ to 41^st^ in Rab39a were indispensable for this interaction. These results suggest that Rab39a negatively regulates the LPS-induced autophagy in macrophages.

## Introduction

Rab GTPases localize to specific subcellular organelles and regulate various membrane trafficking [1,2]. The roles of Rab GTPases in endcytosis and exocytosis have been well studied and their functions in phagocytosis and autophagy are also being elucidated [3-6]. Recently, we screened and identified Rab GTPases that regulate phagosome maturation in macrophages [7]. One of these Rab GTPases, Rab39a was involved in phagosomal acidification [7]. Rab39a has been also demonstrated to be involved in the regulation of Caspase-1 activity [8] and the differentiation of neuron cells [9]. These results together indicate that Rab39a has pleiotropic functions in phagosome maturation, inflammatory activation and neuritogenesis.

Toll-like receptors (TLRs) are pattern recognition receptors to detect infection by recognizing the conserved pathogen-associated molecular patterns such as lipopolysaccharide (LPS) and trigger innate immune responses to defend invading microbes [10,11]. However, if these innate immune responses are dysregulated, it can lead to adverse systemic disorder, sepsis syndrome. Sepsis syndrome is mainly caused by excess inflammatory cytokines secreted from monocytes/macrophages [12]. New immunotherapeutic agents that modulate immune responses have been developing to control sepsis syndrome [13].

In macrophages, the stimulation by LPS or other TLR ligands induces a unique lysosomal degradation pathway for cytoplasmic materials termed autophagy [14-19]. Autophagy induced by LPS contributes to control the inflammatory immune response because protein degradation by autophagy regulates the secretion of inflammatory cytokines [20]. Autophagy pathway is also triggered by invasion of intracellular pathogen and contributes to the protection of host cells [21].

In this study, we characterized the functions of Rab39a in membrane trafficking, phagocytosis and autophagy induction in macrophages. We found that Rab39a interacts with class III phosphatidylinositol 3-kinase (PI3K) and regulates autophagy induced by various TLR stimulations. These results imply the possibility that Rab39a is a potent target molecule in the clinical therapy for the sepsis syndrome.

## Materials and Methods

### Ethics statement

Animal experiments in this study were approved by the Hamamatsu University School of Medicine Animal Care Committees at the Center Animal Care facility (permit number: 2012074). Mice were sacrificed by cervical dislocation and all efforts were made to minimize suffering.

### Cells

Raw264.7 and HEK293T cells were obtained from ATCC and maintained in Dulbecco’s modified Eagle’s medium (DMEM) supplemented with 10% fetal bovine serum (FBS), 25 μg/ml penicillin G and 25 μg/ml streptomycin. Bone marrow-derived macrophages (BMM) were differentiated from bone marrow cells of C57BL/6 mice by culturing in DMEM supplemented with 30% L929-conditional medium, 10% FBS and above antibiotics.

### Plasmid construction

Construction of the plasmid for EGFP-Rab39a was described previously [7]. PCR for Beclin1, Vps34, Atg14L, Rab39b or Bcl2 was carried out using random-primed cDNA derived from human peripheral blood mononuclear cells (Clonetech) as a template and the primer sets listed in Table S1. For UVRAG, PCR was carried out using pCI-HA-UVRAG [22] as a template. PCR products were inserted into pCMV-Myc (Clonetech) or pEGFP-C1 (Clonetech). pEGFP-Rab39a_M1 or pEGFP-Rab39b_M1 was generated by PCR using primers listed in Table S1 and pEGFP-Rab39a or pEGFP-Rab39b as a template, respectively. The resulting PCR products were incubated with T4 DNA ligase and *Dpn* I, followed by the transformation. Transfection of Raw264.7 or HEK293T with plasmid was performed using an MP-100 electroporator (Digital Bio Technology) or X-tremeGENE 9 (Roche), respectively, according to the manufacturer’s instructions.

### Antibody

Rat anti-mouse LAMP2 monoclonal antibody (SouthernBiotech), rabbit anti-LC3 polyclonal antibody (MBL), mouse anti-tubulin monoclonal antibody (Sigma-Aldrich), rabbit anti-p62 antibody (MBL), mouse anti-ubiquitin antibody (MBL), rabbit anti-Beclin1 antibody (MBL), rabbit anti-GM130 antibody (MBL), mouse anti-EGFP monoclonal antibody (Clonetech), rat anti-EGFP monoclonal antibody (Nacalai tesque) , mouse anti-c-Myc antibody (Wako), rabbit anti-Vps34 antibody (CST), mouse anti-UVRAG antibody (MBL) and rabbit anti-ATG14L antibody (MBL) were used as primary antibodies. Alexa488- or Alexa546-conjugated anti-IgG antibodies (Invitrogen) and horseradish peroxidase-conjugated anti-IgG antibodies (Dako) were used as secondary antibodies.

### Fluorescence and thin-section electron microscopy

Immunofluorescence and thin-section electron microscopic analyses were performed as described previously [23,24]. For immunofluorescence microscopy, macrophages were stained with anti-LAMP2 antibody (1:25 v/v), anti-LC3 antibody (1:25 v/v), anti-p62 antibody (1:25 v/v), anti-ubiquitin antibody (1:25 v/v), anti-Beclin1 (1:25 v/v) or anti-GM130 (1:10 v/v).

### Fluorescence recovery after photobleaching analysis

Fluorescence recovery after photobleaching (FRAP) analysis was performed as described previously [25]. Briefly, transfected cells with plasmid expressing EGFP-Rab39a (3 × 10^5^ cells) grown on 35-mm glass dishes were allowed to phagocytose latex beads for 2 h. FRAP analysis was performed using an FV1000-D confocal microscope (Olympus) with a 60×/1.4 numerical aperture oil-immersion objective lens. The area of the phagosome surrounded by EGFP-Rab39a was photobleached using a 410-nm laser at 15–20% power for 300 ms after pre-bleached images were acquired. Following photobleaching, the recovery of fluorescence was monitored at every 2 sec using a 488-nm laser at 1-2 % power for the phagosomes containing latex beads. Fluorescence intensities were quantified using the ImageJ (http://rsb.info.nih.gp/ij/).

### RNA interference

siRNA duplexes were synthesized by Sigma-Aldrich as listed in Table S2. siRNA designated as Rab39a#2 is an siGENOME SMART pool of Mouse Rab39 purchased from Thermofisher Science. The sequence of siRNA for Atg5 was described previously [26]. Mission siRNA universal negative control (Sigma-Aldrich) was used as control siRNA. Transfection of macrophages with siRNA duplexes were performed using Lipofectamine RNAiMAX (Invitrogen) according to the manufacturer’s instructions. Real-time quantitative-PCR (RT-qPCR) was performed using SYBR Premix Ex Taq (TaKaRa) and the primer sets listed in Table S3.

### Immunoblot and immunoprecipitation analyses

For immunoblot (IB) analysis, macrophages or HEK293T cells were extracted with 8 M urea or immunoprecipitation (IP) buffer containing 150 mM NaCl, 50 mM Tris-HCl (pH 7.5), 1 mM EDTA, and 1% Triton X-100, 1 mM phenylmethanesulfonyl fluoride, 1 mM Na_3_VO_4_, and protease inhibitor cocktail (Roche), respectively. Cell lysates were separated by SDS-polyacrylamide gel electrophoresis (SDS-PAGE) and then subjected to immunoblot analysis using anti-LC3 antibody (1:500 v/v), anti-tubulin antibody (1:1000 v/v), anti-p62 antibody (1:500 v/v), anti-c-Myc antibody (1:1000 v/v) and mouse anti-EGFP antibody (1:1000 v/v).

IP analysis was performed as described previously [22]. For IP of GFP, aliquots of 500 μl of cell lysates containing 500 μg of proteins were immunoprecipitated by using 1 μg of rat anti-EGFP antibody. For IP of endogenous Beclin1, Vps34, UVRAG or Atg14L, an aliquot of 2 μg of the antibody against each protein was used.

### Statistics

The paired or unpaired two-sided Student’s *t*-test was used to assess the statistical significance of differences between the two groups. To assess the proportions of cells with LC3-dot or Beclin1-dot, three or four independent experiments were conducted, and more than 200 cells were counted for each condition. Fluorescence with more than 1 μm-diameter was judged as LC3-dot or Beclin-1 dot.

## Results

### Rab39a localizes to lysosomes

**Figure 1:**
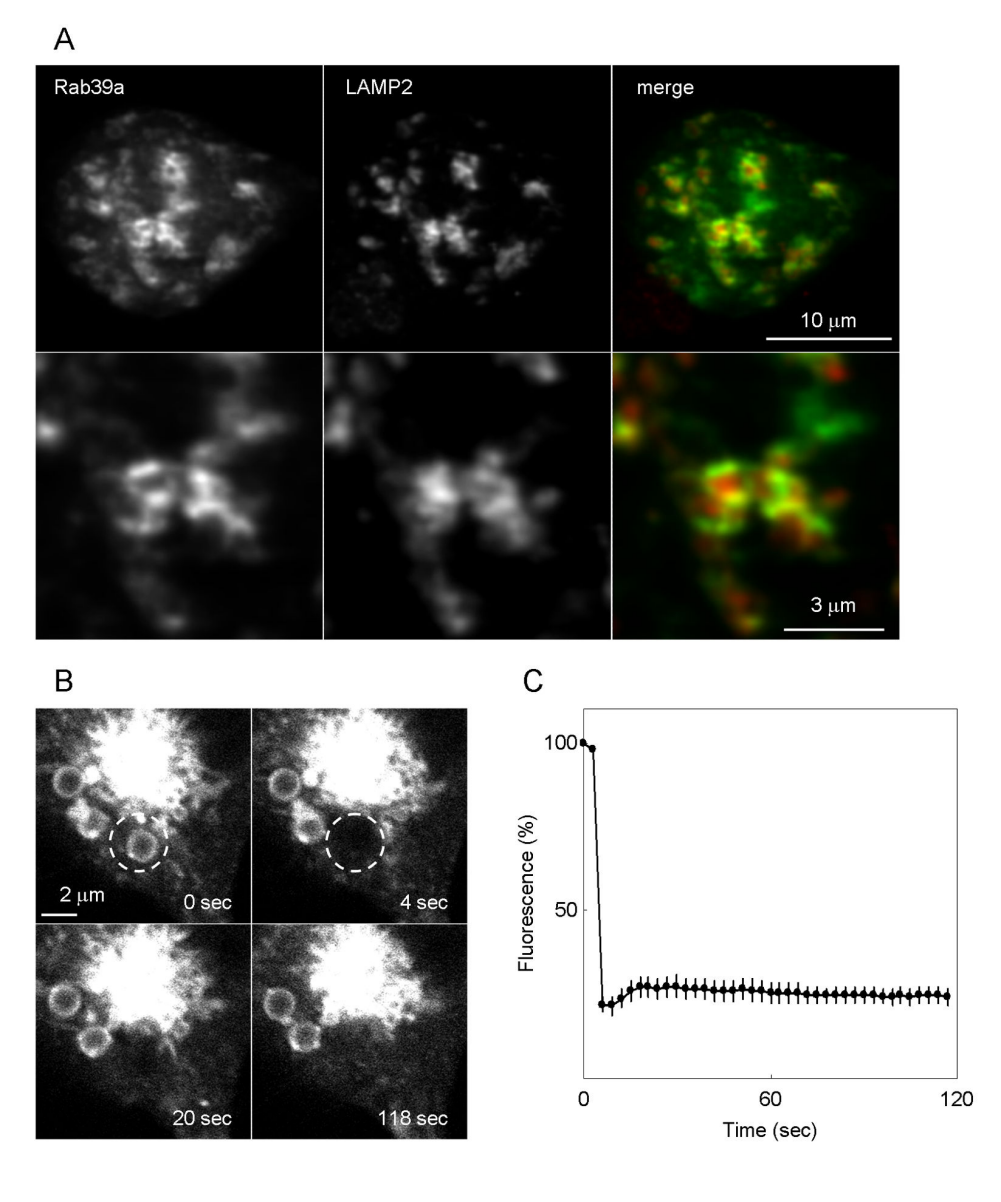
Localization of Rab39a in macrophages. (A) Immunostaining of Raw264.7 macrophages expressing EGFP-Rab39a with anti-LAMP2 antibody. (B) Representative sequence images from FRAP analysis of EGFP-Rab39a on latex bead-containing phagosomes. The region marked by a broken-line circle was photobleached at 4 sec, and the recovery of fluorescence was monitored. (C) Temporal changes in fluorescence intensities on the bleached phagosomes. The relative intensity was defined as the ratio of fluorescence intensity at each time point to that at 0 sec. Data represent means and standard errors of means (n=10).

To characterize the localization of Rab39a in macrophages, Raw264.7 macrophages were transfected with EGFP-Rab39a and immunostained with anti-LAMP2 antibody. Rab39a specifically localized to the periphery of LAMP2-positive organelles in the steady state condition (Figure 1A and Figure S1A). In phagocytosis, Rab39a co-localizes with LAMP2 on *E. coli*-containing phagosomes (Figure S1B). To assess the motility of Rab39a on the phagosome, we next conducted FRAP analysis on the latex bead-containing phagosomes in Raw264.7 macrophages expressing EGFP-Rab39a. By FRAP analysis, we can assess the state of activation of Rab39a on the membrane, because the active GTP-bound forms of Rab GTPases are stable on the membrane while the inactive GDP-bound forms release from the membrane to the cytosol. After the photobleaching of the phagosomes with EGFP-Rab39a (Time 4 sec), fluorescence recovery was very weak (Figure 1B and Movie S1). Quantitative analysis revealed that the recovery rate of fluorescence is 14% of the original level and the recovery half time for fluorescence is 7.1 sec (Figure 1C). The kinetic of Rab39a on the phagosomes was similar to that of LAMP1 [25]. These results suggest that Rab39a is a component protein of lysosomal vesicles and resides on the phagosomal membrane.

### Rab39a depletion does not influence the trafficking of LAMP2 to phagosomes

Since Rab39a functions in the acidification of phagosomes [7], we hypothesized that Rab39a regulates the fusion of lysosomes with phagosomes. To address the function of Rab39a in phagolysosome biogenesis, we transfected Raw264.7 macrophages with siRNA for Rab39a and first confirmed the decrease of mRNA for Rab39a induced by the transfection (Figure S2A, B). We next examined the trafficking of LAMP2 to *E. coli*-containing phagosomes in these Rab39a-knockdown (KD) macrophages. LAMP2 localized to the phagosomes in Rab39a-KD macrophages as in control macrophages (Figure S2C). Quantitative analysis revealed that the proportion of LAMP2-positive phagosomes in Rab39a-KD macrophages is not significantly different from that in control macrophages (Figure S2D). These results suggest that Rab39a depletion does not influence the localization of LAMP2 to the phagosome.

### Rab39a depletion augments autophagy induced by LPS

**Figure 2:**
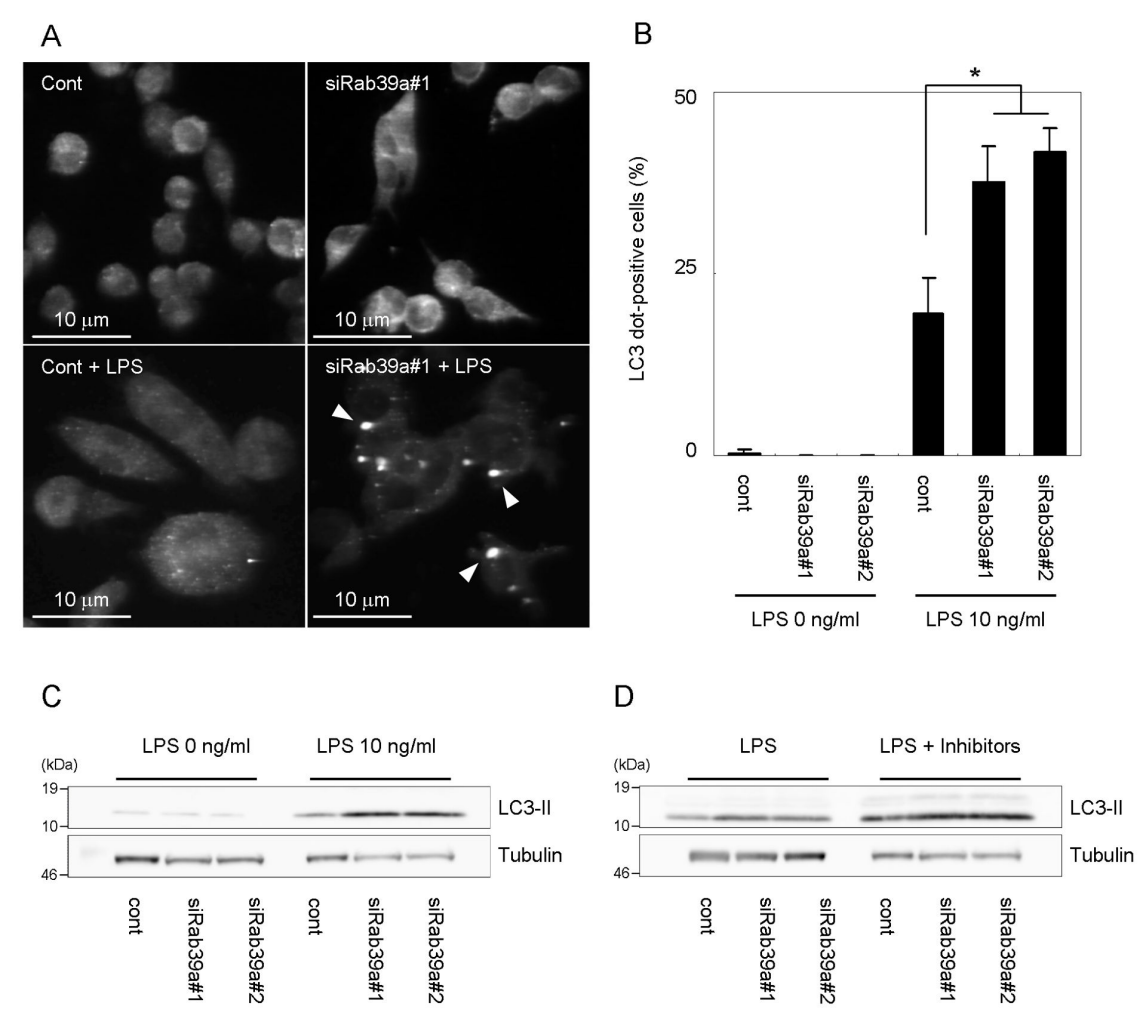
Augmentation of autophagy induced by LPS in Rab39a-KD macrophages. (A) Immunostaining analysis of LPS-induced autophagy in Rab39a-KD macrophages. Raw264.7 macrophages transfected with control or Rab39a siRNA were treated with LPS for 24 h and immunostained with anti-LC3 antibody. An arrowhead indicates an LC3-dot fluorescence. (B) Proportion of Raw264.7 macrophages with LC3-dots induced by LPS stimulation for 24 h. Data represent the mean and SD of three or four independent experiments. *p < 0.05 (unpaired Student’s *t*-test). (C) Immunoblot analysis of LC3 processing in macrophages treated with LPS. Raw264.7 macrophages transfected with control or Rab39a siRNA were treated with LPS for 24 h and subjected to immunoblot analysis using indicated antibodies. (D) Autophagic flux in Rab39a-KD macrophages treated with LPS. Raw264.7 macrophages treated with control or Rab39a siRNA were treated with LPS in the absence or presence of protease inhibitors, E64d and pepstatin A, for 24 h. Cell lysates were subjected to immunoblot analysis using indicated antibodies.

Because we found that the infection of *E. coli* induces the formation of LC3 punctuation in Rab39a-KD macrophages by immunofluorescence microscopy (data not shown), we examined the effect of Rab39a depletion on autophagy induction. To investigate the effect of Rab39a depletion on classical autophagy pathway, we treated Rab39a-KD macrophages with rapamycin and found that Rab39a depletion does not influence autophagy induced by rapamycin (Figure S3). LPS stimulation is also reported to induce autophagy in macrophages [14,18,19]. Therefore, we examined the autophagosome formation and LC3 processing induced by LPS in Rab39a-KD macrophages (Figure 2). Immunofluorescence microscopic analysis revealed that the formation of LC3-positive aggregations induced by LPS is augmented in Rab39a-KD macrophages (Figure 2A, B). Thin-section electron microscopic analysis also demonstrated that the LPS stimulation induces the formation of electron dense-aggregation in Rab39a-KD macrophages (Figure S4A) as reported previously [15,27]. Immunoblot analysis showed that LPS stimulation augments the processing of LC3 in Rab39a-KD macrophages comparing with that in control macrophages (Figure 2C). In BMM, depletion of Rab39a again augmented the formation of the LC3-positive aggregation and processing of LC3 in response to LPS stimulation (Figure S5). To evaluate the lysosomal-dependent autophagic degradation (autophagy flux), we treated Raw264.7 macrophages with LPS in the presence of protease inhibitors, E64d and pepstatin A, and examined the processing of LC3 (Figure 2D). Quantitative analysis revealed that the treatment with protease inhibitors augmented the processing of LC3 in response to LPS stimulation in Rab39a-KD macrophages as well as in control macrophages (Figure S6A). These results suggest that Rab39a depletion does not influence autophagy flux. We also examined the co-localization of LC3 or p62/ SQSTM1 (p62) with ubiquitin in Rab39a-KD macrophages stimulated by LPS and found that both LC3 and p62 co-localize with ubiquitin-positive aggregation (Figure S4B), suggesting that depletion of Rab39a augments the formation of LPS-induced selective autophagosome in macrophages.

**Figure 3:**
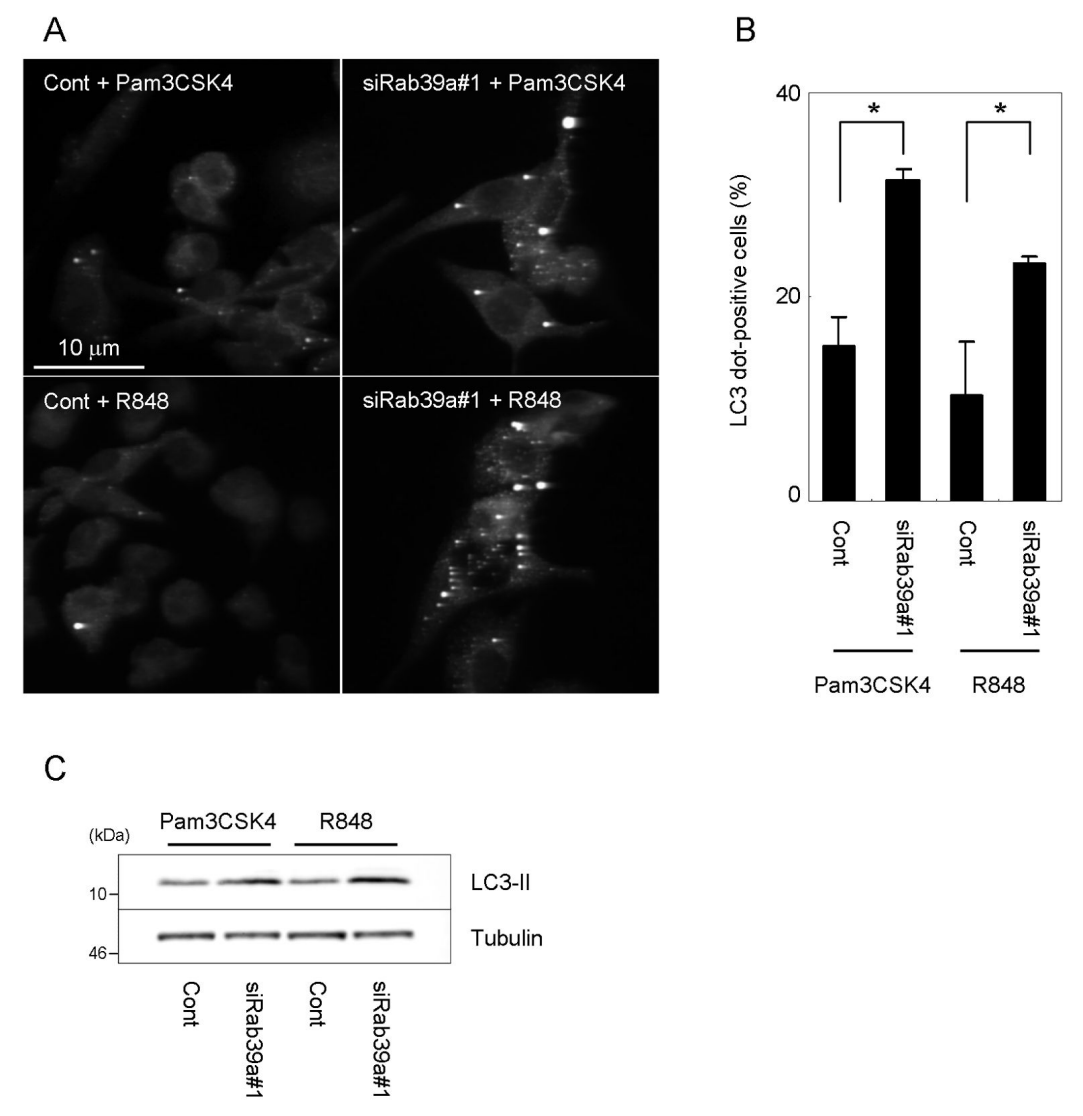
Autophagy induced by TLR2 or TLR7/8 ligand in Rab39a-KD macrophages. (A) Immunostaining analysis of Rab39a-KD macrophages stimulated by TLR2 or TLR7/8 ligand. Raw264.7 macrophages transfected with control or Rab39a siRNA were treated with TLR2 ligand Pam3CSK4 at 10 ng/ml or TLR7 ligand R848 at 1 μg/ml for 24 h and immunostained with anti-LC3 antibody. (B) Proportion of Raw264.7 macrophages with LC3-dots induced by Pam3CSK4 or R848 for 24 h. (C) Immunoblot analysis of LC3 processing in macrophages treated with Pam3CSK4 or R848. Raw264.7 macrophages transfected with control or Rab39a siRNA were treated with Pam3CSK4 or R848 for 24 h and subjected to immunoblot analysis using indicated antibodies. Data represent the mean and SD of three or four independent experiments. *p < 0.05 (unpaired Student’s *t*-test).

We next addressed the augmentation effect of Rab39a depletion in autophagy induced by other TLR ligands (Figure 3). We found that Pam3CSK4 (for TLR2) and R848 (for TLR7/8) induce the autophagosome formation in macrophages as described previously [14,19]. The depletion of Rab39a augmented the autophagosome formation and the processing of LC3 by stimulation of Pam3CSK4 or R848 (Figure 3A-C). These results altogether indicate that Rab39a depletion also augments autophagy induced by other TLR ligands.

**Figure 4:**
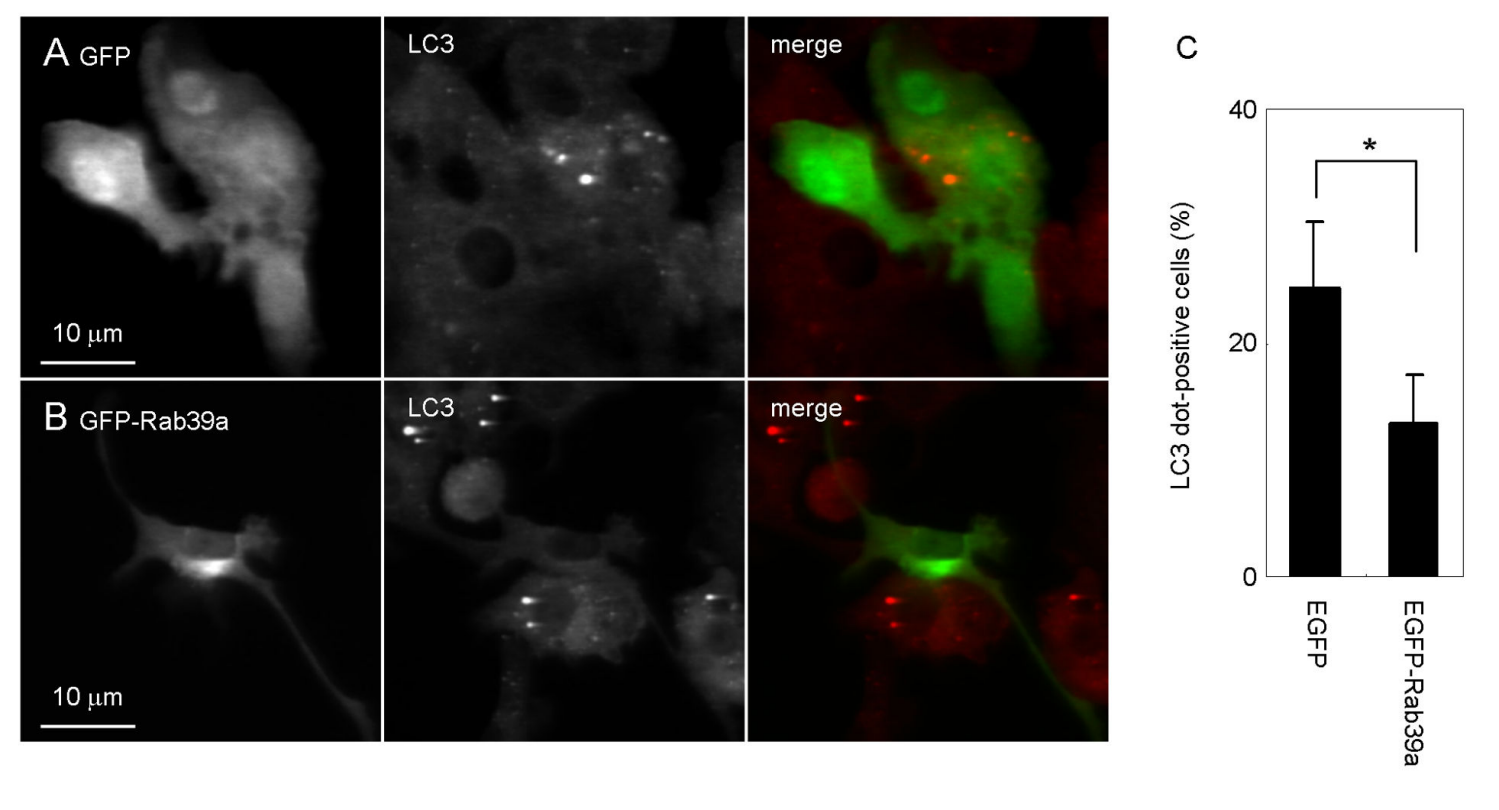
Suppression of LPS-induced autophagosome formation by Rab39a overexpression. (A, B) Raw264.7 macrophages expressing EGFP (A) or EGFP-Rab39a (B) were treated with LPS and immunostained with anti-LC3 antibody. (C) Proportion of macrophages with LC3-dot expressing EGFP or EGFP-Rab39a induced by LPS stimulation for 24 h. Data represent the mean and SD of three independent experiments. *p < 0.05 (unpaired Student’s *t*-test).

We examined the effect of overexpression of Rab39a in LPS-induced autophagy (Figure 4). Macrophages expressing EGFP or EGFP-Rab39a were treated with LPS and autophagosome formation was examined by immunofluorescence microscopy. The formation of LC3-dot decreased in macrophages expressing EGFP-Rab39a, suggesting that Rab39a overexpression suppresses the LPS-induced autophagy. Taken together, these results suggest that Rab39a negatively regulates the autophagy induced by TLR ligands in macrophages.

### Mechanism of autophagy augmentation induced by LPS in Rab39a-KD macrophages

**Figure 5:**
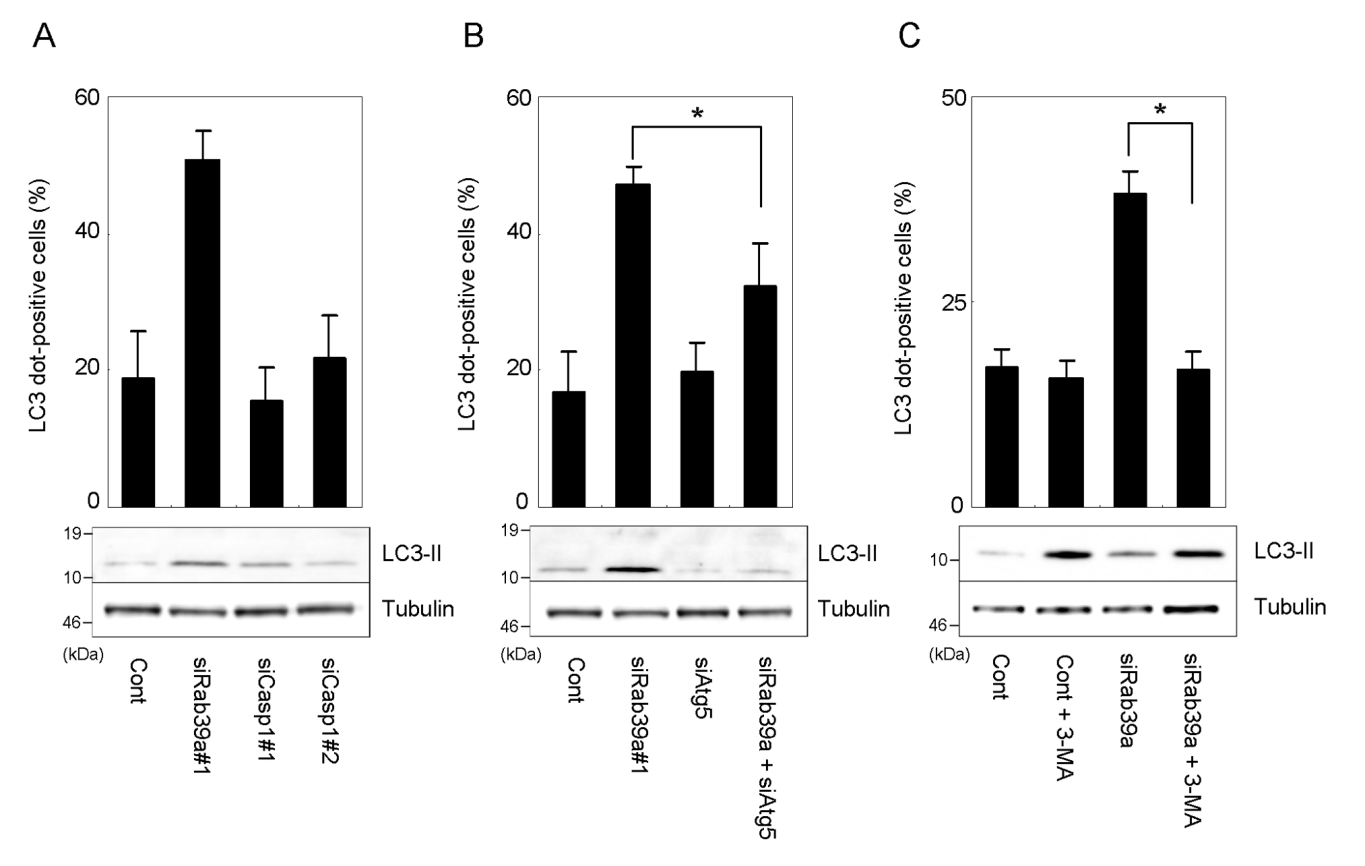
Involvement of Caspase-1 and classical autophagy pathway in autophagy induced by LPS (A) Autophagy induced by LSP in Caspase-1-KD macrophages. The proportion of macrophages with LC3-dot (upper panel) and immunoblot analysis of LC3 processing (lower panel) are shown. (B) Autophagy induced by LSP in Atg5-KD macrophages. The proportion of macrophages with LC3-dot (upper panel) and immunoblot analysis of LC3 processing (lower panel) are shown. (C) Autophagy induced by LPS in the presence of a PI3K inhibitor, 3-MA. Transfected macrophages were stimulated by LPS in the presence or absence of 3-MA at 10 mM for 24 h. The proportion of macrophages with LC3-dot (upper panel) and immunoblot analysis of LC3 processing (lower panel) are shown. Data represent the mean and SD of three or four independent experiments. *p < 0.05 (unpaired Student’s *t*-test).

Since Rab39a binds Caspase-1 and regulates its activity [8], it is possible that Rab39a-bound Capsase-1 regulates the autophagy induced by LPS. We examined the effect of Caspase-1 depletion on autophagosome formation and LC3 processing in LPS-stimulated Raw264.7 macrophages and found no effect of Caspase-1 depletion on these events (Figure 5A). We also examined the expression level of p62 in Rab39a-KD macrophages, because the increase of p62 expression plays the pivotal role in LPS-induced autophagy [15]. However, immunoblot analysis revealed that the expression level of p62 in LPS-stimulated Rab39a-KD macrophages does not significantly change comparing with that in LPS-stimulated control macrophages (Figure S7).

We examined the autophagy induction in macrophages transfected with siRNA for Atg5 (Figure 5B). Atg5 depletion decreased LC3 processing but did not influence the autophagosome formation induced by LPS stimulation as reported previously [15]. We found that Atg5 depletion suppressed the augmentation of autophagy induction by LPS in Rab39a-KD macrophages. These results suggest that Rab39a regulates the classical autophagic pathway activated by LPS stimulation.

Poteomic analysis demonstrated the interaction of Rab39a with a class III PI3K [28] that regulates autophagy induction [29]. To investigate whether PI3K activation is involved in augmentation of autophagy induced by LPS in Rab39a-KD macrophages, we treated transfected macrophages with a PI3K inhibitor, 3-methyladenine (3-MA) [30] and examined the autophagosome formation and LC3 processing in response to LPS (Figure 5C). 3-MA treatment increased LC3 processing in both control and Rab39a-KD macrophages stimulated by LPS. This result is consistent with the previous report that prolonged 3-MA treatment increases LC3 processing [31]. However, 3-MA treatment decreased the autophagosome formation induced by LPS stimulation in Rab39a-KD macrophages but not in control mcrophages, suggesting that the PI3K activation is involved in this augmentation.

**Figure 6:**
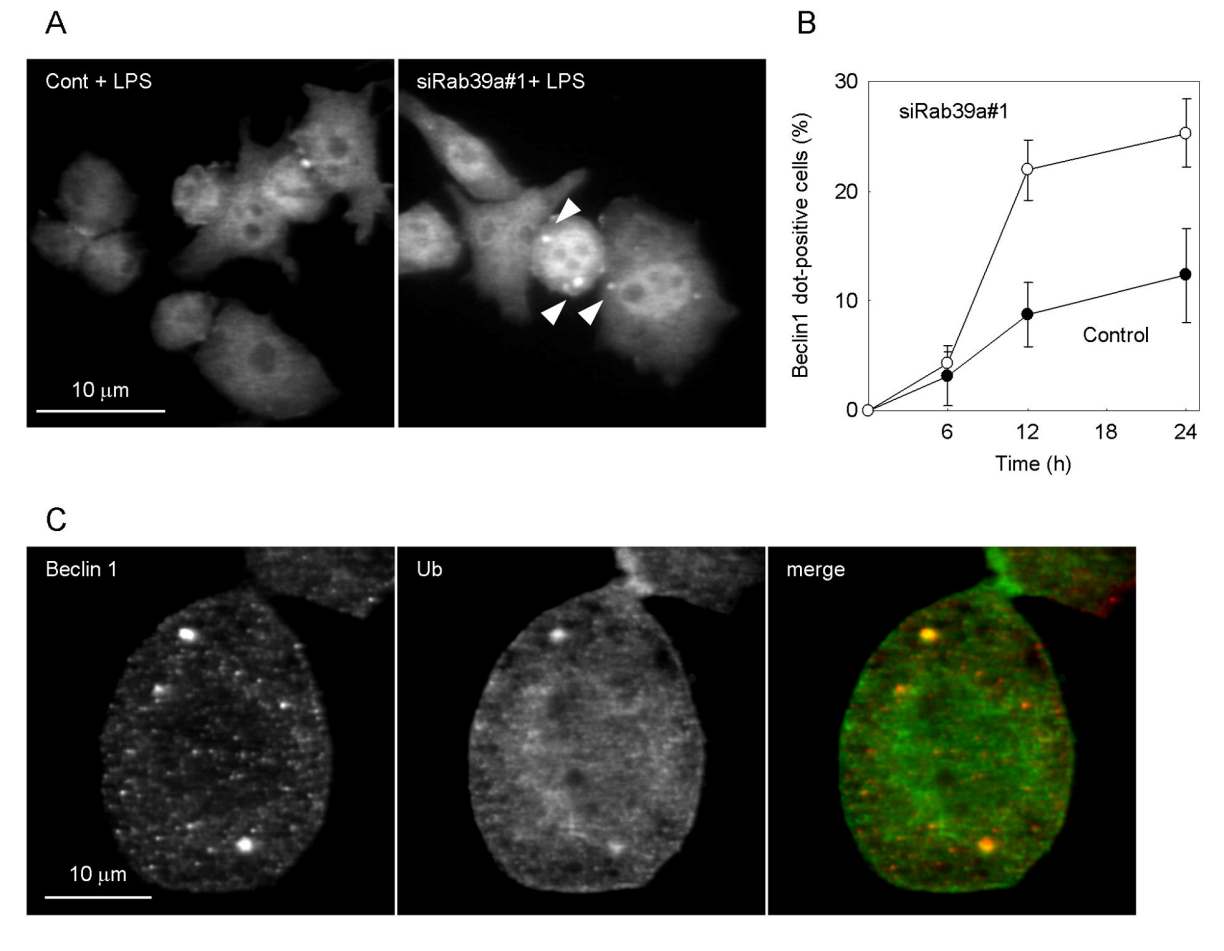
Beclin1-dot formation in LPS-induced autophagy. (A) Control or Rab39a-KD macrophages were treated with LPS for 12 h and immunostained with anti-Beclin1 antibody. An arrowhead indicates a Beclin1-dot. (B) The proportion of control or Rab39a-KD macrophages with Beclin-dot in LPS-induced autophagy is shown. Data represent the mean and SD of three independent experiments. (C) Localization of Beclin1-dot. Rab39a-KD macrophages were treated wtih LPS for 12 h and immunostained with anti-Beclin1 (red) and anti-ubiquitin (green) antibodies.

We addressed the Beclin1 localization in LPS-treated Rab39a-KD macrophages (Figure 6). Beclin1 interacts with PI3K and regulates the initiation of autophagy [32]. Rab39a depletion promoted the formation of Beclin1-dots during LPS-induced autophagy (Figure 6A, B), suggesting that LPS stimulation induces the formation of PI3K complex in Rab39a-KD macrophages. We also found that Beclin1 localized to ubiquitin aggregation (Figure 6C), suggesting that PI3K localizes to autophagosome in LPS-induced autophagy in Rab39a-KD macrophages. These results altogether suggest that augmentation of LPS-induced autophagy in Rab39a KD macrphages is caused by the increase of PI3K activity.

### Rab39a interacts with PI3K

**Figure 7:**
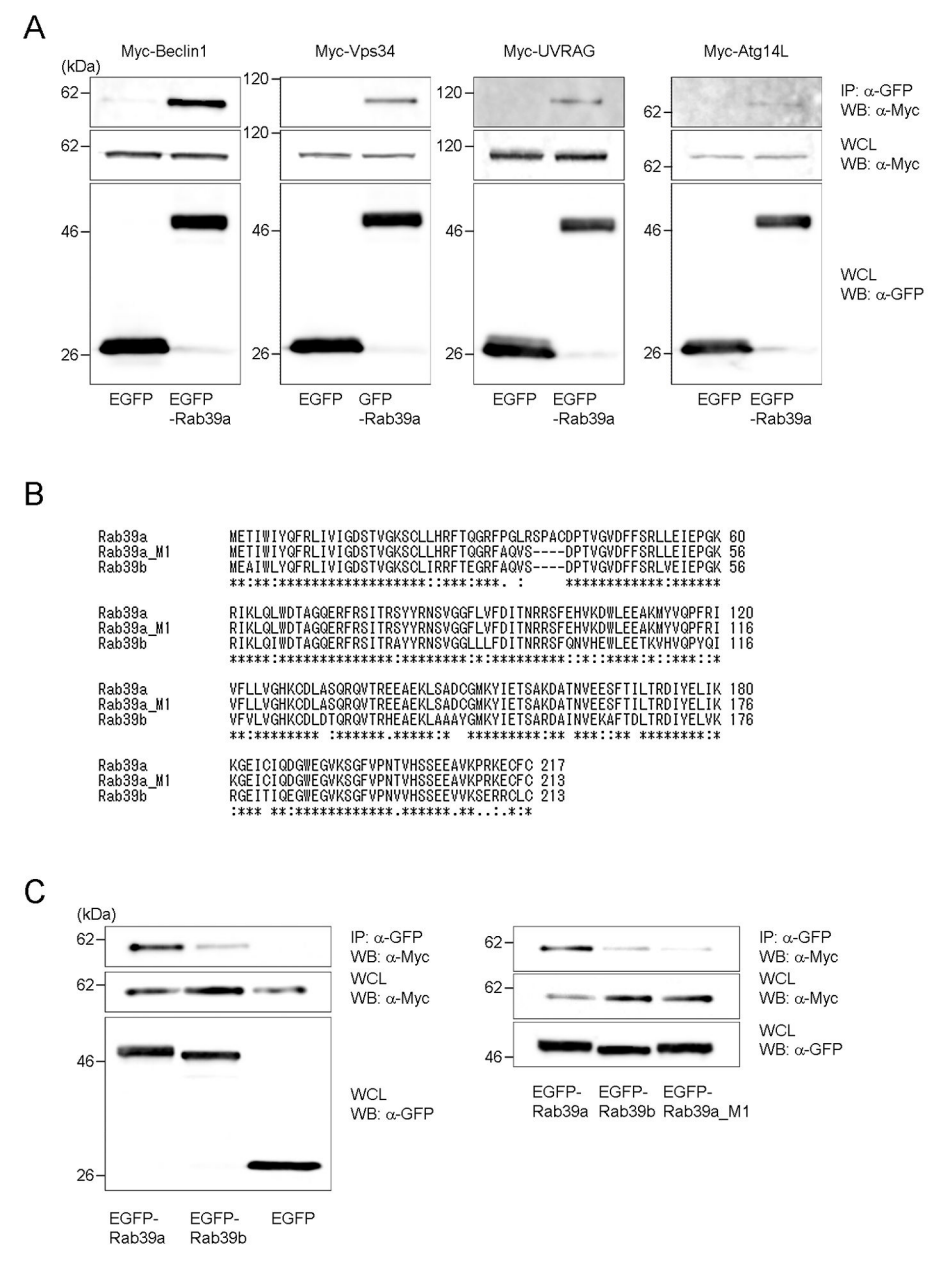
Interaction of Rab39a with Beclin1. (A) HEK293T cells were transfected with plasmids for Myc-Beclin1, Myc-Vps34, Myc-UVRAG or Myc-Atg14L and EGFP-Rab39a or EGFP. Whole cell lysates (WCL) were used for immunoprecipitation (IP) with anti-GFP antibody, followed by immunoblot analysis (IB) with anti-Myc antibody. For detection of input, aliquots of 15 μg of WCL were used. (B) ClustalW alignment of the amino acid sequences of Rab39a, Rab39a_M1 and Rab39b is shown. (C) HEK293T cells were transfected with plasmids for Myc-Beclin1 and EGFP-Rab39a, EGFP-Rab39a_M1, EGFP-Rab39b or EGFP. WCL were used for IP with anti-GFP antibody followed by IB with anti-Myc antibody. For detection of input, aliquots of 15 μg of WCL were used.

We finally examined the interaction of Rab39a with components of PI3K for autophagy regulation (Figure 7A). Rab39a co-immunoprecipitated with Beclin1, Vps34, UVRAG and Atg14L in HEK293T cells, confirming that Rab39a interacts with PI3K as reported previously [28]. Quantification analysis revealed that Rab39a preferentially co-precipitates with Beclin1 (Figure S6B). We also found that endogenous Beclin1, not Vps34, UVRAG or Atg14L interact with Rab39a (Figure S8A and data not shown), suggesting that Rab39a interact with PI3K complex via Beclin1. Bcl2 interact with Beclin1 and regulates the PI3K activity [32]. We examined the interaction of Rab39a with Bcl2 and found little association (Figure S8B). Rab39b has a high sequence similarity with Rab39a and localizes to the Golgi complex [33]. However, amino acid sequence alignment showed that Rab39b lacks the amino acid residues from 34^th^ to 41^st^ of Rab39a (Figure 7B). Therefore, we constructed Rab39a_M1 that has the same amino acid residues as found in Rab39b at this region and assessed the effects of this conversion. We found that Rab39a_M1 shows the similar localization of Rab39b in macrophages (Figure S9) and localization of both proteins was different from that of Rab39a (Figure S10); Rab39a_M1 and Rab39b did not specifically localize to the periphery of LAMP2-positive organelles but cover these organelles. We examined the interaction of Rab39a_M1 with Beclin1 by IP analysis and found that Rab39a_M1 and Rab39b show weaker interaction with Beclin1 than Rab39a (Figure 7C). Furthermore, we examined the interaction of Rab39b_M1, which has the amino acid residues from 34^th^ to 41^st^ of Rab39a, with Beclin1 (Figure S11). Quantitative analysis revealed that Rab39b_M1 shows the stronger interaction with Beclin1 than Rab39b. These results suggest that the amino acid residues from 34^th^ to 41^st^ in Rab39a are important for its interaction with PI3K.

## Discussion

Rab GTPases are conserved throughout eukaryotes and regulate the membrane trafficking of intracellular processes. Rab39 is also conserved in non-vertebrate and vertebrate animals. In *Caenorhabditis elegans*, Rab39 homolog is involved in oxidative stress response [34]. In *Drosophila*, Rab39 homolog functions in lipid storage in adipose tissue [35]. In mammal, Rab39a is shown to be involved in distinct cellular processes [7-9]. In this study, we performed the detail analyses in the function of Rab39a in macrophages and found that Rab39a regulates autophagy induced by LPS stimulation.

Rab39a localized to the periphery of LAMP2-positive vesicles (Figure 1) and was involved in the phagosomal acidification in macrophages [7]. FRAP analysis revealed that Rab39a is active form and resides on the phagosome membrane (Figure 1), suggesting that Rab39a interacts with effector proteins to recruit on the phagosomal membrane and regulate acidification during phagolysosome biogenesis. However, we also found that LAMP2 localized to *E. coli*-containing phagosomes in Rab39a-KD macrophages. Because Huynh et al reported that LAMP1/2 localize to the phagosome before Rab7 [36], the trafficking of LAMP1/2 to the phagosome is supposed to precede that of Rab39a.

We investigated the mechanism by which Rab39a depletion augments the autophagy in response to LPS. Rab39a is reported to interact with Caspase-1 and regulate its activity [8]. Since inflammatory cytokines including IL-1β and IL-18 regulate autophagy [37], it is possible that the decrease of Caspase-1 activity in macrophages influences the induction of autophagy. However, we found that Caspase-1 depletion does not mediate the augmentation of autophagy induced by LPS (Figure 5A). We also found that Caspase-1 inhibitor Z-YVAD-FMK does not influence the autophagosome formation induced by LPS (data not shown). These results suggest that the Caspase-1 activity is not involved in regulation of autophagy induced by LPS in macrophages.

TLR stimulation activates nuclear factor (NF)-κB [38,39] that is reported to bind to promoter of Beclin1 and up-regulate its expression resulting in autophagy induction [40]. These results suggest that autophagy induced by TLR stimulation is caused by up-regulation of the classical autophagic pathway through NF-κB activation. On the contrary, it is reported that the classical autophagy pathway is dispensable for autophagy induced by LPS, but up-regulation of p62 expression in response to LPS stimulation plays the pivotal role in this autophagy [15]. In this study, we found that Atg5 depletion suppresses the effect of Rab39a depletion on LPS-induced autophagy (Figure 5B), whereas Rab39a depletion itself does not influence the classical autophagy induction (Figure S3). A specific PI3K inhibitor, 3-MA, suppressed the autophagosome formation by LPS stimulation in Rab39a-KD but not control macrophages (Figure 5C). These results suggest that LPS stimulation activates the classical autophagy pathway and that Rab39a prevents its signaling.

Beclin1-Vps34 complex regulates distinct membrane trafficking pathways by interacting with different regulatory proteins [32,41]. It is also demonstrated that UVRAG or Atg14L interacts with Beclin1 to exclude the other protein from its complex [22,42-44]. Rab7 interacts with PI3K to regulate endocytosis pathway and autophagosome maturation [45-47]. In this study, we found that Rab39a also interacts with Beclin1-Vps34 complexes (Figure 7). We also found that UVRAG and Atg14L are immunoprecipitated with Rab39a, suggesting that Rab39a does not interfere the interaction of UVRAG or Atg14L with Beclin1. Rab39a and Rab39b have highly sequence similarities, but their subcellular localization is different; Rab39a and Rab39b localizes to lysosomes (Figure 1) and the Golgi complex [33], respectively. We examined the localization of Rab39a and Rab39b with GM130, a Golgi-marker protein [48] in Raw264.7 macrophages and found that Rab39b overlaps where GM130 localizes, whereas Rab39a shows the localization distinct from this region (Figures S9 and S10). We also found that Rab39a interacts with Beclin1 at the specific sequences lacked in Rab39b (Figure 7). Rab39a mutant lacking this region showed similar localization of Rab39b and impairment of its interaction with Beclin1 (Figures S9 and S10). These results suggest that the unique sequence of Rab39a responsible for the interaction with Beclin1 is also involved in Rab39a localization to the periphery of lysosomes.

In summary, our results suggest that TLR stimulation induces autophagy via the activation of two distinct pathways, i.e., increase of p62 expression and activation of class III PI3K. Rab39a could interact with PI3K and negatively regulate its activity in induction of autophagy by TLR stimulation. Therefore, Rab39a depletion resulted in the augmentation this type of autophagy. Our results also imply the possibility that Rab39a is a potent target molecule to control the sepsis syndrome because Rab39a regulates the inflammatory responses by controlling Caspase-1 activity and autophagy induction in response to LPS stimulation.

## Supporting Information

Figure S1 **Rab39a localizes to lysosomes.** (A) Projection of focal planes with y-z and x-z side views of Figure 1A is shown. (B) Localization of Rab39a in macrophages infected with *E. coli*. Raw264.7 macrophages expressing EGFP-Rab39a were infected with Alexa-405 labeled *E. coli* for 2 h and immunostained with anti-LAMP2 antibody. An arrowhead indicates a Rab39a-positive, LAMP2-positive *E. coli*-containing phagosome.
(TIF)

Figure S2 **LAMP2 localization to phagosomes in Rab39a-KD macrophages.** (A) RT-PCR analysis of Raw264.7 macrophages transfected with siRNA duplexes for Rab39a. (B) Quantitative analysis of Rab39 transcription. Rab39a mRNA level was assessed by RT-qPCR using GAPDH mRNA as a control. (C) Localization of LAMP2 to *E. coli*-containing phagosomes in Rab39a-KD macrophages. Raw264.7 macrophages transfected with siRNA for Rab39a were incubated with TexasRed-labeled *E. coli* for 2 h and were immunostained with anti-LAMP2 antibody. (D) The proportion of LAMP2-positive phagosomes in Rab39a-KD macrophages. Data represent the mean and SD of three independent experiments. *p < 0.05 (unpaired Student’s *t*-test).
(TIF)

Figure S3 **Rapamycin-induced autophagy in Rab39a-KD macrophages.** Raw264.7 macrophages transfected with control or Rab39a siRNA were treated with rapamycin at 50 μg/ml for 2 h. (A) Cells were fixed and immunostained with anti-LC3 antibody. (B) Cell extracts were subjected to immunoblot analysis for anti-LC3 antibody.
(TIF)

Figure S4 **Characterization of LPS-induced autophagosome formation in Rab39a-KD macrophages.** (A) Thin-section electron micrograph of Rab39a-KD macrophages. Raw264.7 macrophages transfected with control or Rab39a siRNA were treated with LPS at 10 ng/ml for 24 h and observed by thin-electron microscopy. An arrowhead indicates the electron dense-aggregation. (B) Co-localization of LC3 or p62 and ubiquitin in Rab39a-KD macrophages treated with LPS. Raw264.7 macrophages transfected with Rab39a siRNA were treated with LPS for 24 h and immunostained with anti-LC3 or anti-p62 and anti-ubiquitin antibodies.
(TIF)

Figure S5 **Augmentation of autophagy induced by LPS in Rab39a-KD BMM.** (A) Immunofluorescence analysis of LC3 distribution. BMM transfected with control or Rab39a siRNA were treated with LPS for 24 h and immunostained with anti-LC3 antibody. (B) Immunoblot analysis of LC3 processing in BMM treated with LPS. Raw264.7 macrophages transfected with control or Rab39a siRNA were treated with LPS for 24 h and subjected to immunoblot analysis using indicated antibodies. *, non-specific band.
(TIF)

Figure S6 **(**A**) The quantification of band intensity for LC3-II in Figure 2B is shown.** The ratio of band intensity for LC3-II/tubulin at each condition to that in control macrophages is shown. Data represent the mean and SD of three independent experiments. *p < 0.05 (paired Student’s *t*-test). (B) The quantification of band intensity for immunoprecipitated proteins with EGFP-Rab39a in Figure 7A is shown. The ratio of band intensity of each protein for immunoprecipitation (IP)/whole cell lysate (WCL) to that of Beclin1 is shown. Data represent the mean and SD of three independent experiments. *p < 0.05 (paired Student’s *t*-test).
(TIF)

Figure S7 **Expression level of p62 in Rab39a-KD macrophages.** Raw264.7 macrophages transfected with control or Rab39a siRNA were treated with LPS for 24 h. Cell extracts were subjected to immunoblot analysis for anti-p62 antibody.
(TIF)

Figure S8 **Interaction of Rab39a with Beclin1 or Bcl2.** (A) HEK293T cells were transfected with plasmid for EGFP-Rab39a or EGFP-Rab39b. Whole cell lysates (WCL) were used for immunoprecipitation (IP) with anti-Beclin1 antibody, followed by immunoblot analysis (IB) with anti-GFP antibody. For detection of input, aliquots of 5 μg of WCL were used. (B) HEK293T cells were transfected with plasmids for Myc-Bcl2 and EGFP-Rab39a or EGFP-Rab39b. WCL were used for IP with anti-GFP antibody, followed by IB with anti-Myc antibody. For detection of input, aliquots of 5 μg of WCL were used.
(TIF)

Figure S9 **Subcellular localization of Rab39b and Rab39a_M1 in macrophages.** Raw264.7 macrophages were transfected with the expression plasmid for EGFP-Rab39a, EGFP-Rab39b or EGFP-Rab39a_M1 and immunstained with anti-LAMP2 antibody (A, B) or anti-GM130 antibody (C, D, E).
(TIF)

Figure S10 **Enlarged images of Figures 1A and S5.** Quantification for the fluorescence intensities across the arrows in right panels is also shown.
(TIF)

Figure S11 **Amino acid residues from 34^th^ to 41^st^ of Rab39a is important for interaction of Rab39a with Beclin1.** (A) ClustalW alignment of the amino acid sequences of Rab39a, Rab39b, Rab39a_M1 and Rab39b_M1 is shown. (B) HEK293T cells were transfected with plasmids for Myc-Beclin1 and EGFP-Rab39a, EGFP-Rab39b, EGFP-Rab39a_M1 or EGFP-Rab39b_M1. WCL were used for IP with anti-GFP antibody followed by IB with anti-Myc antibody. For detection of input, aliquots of 15 μg of WCL were used. (C) Quantification of band intensity for immunoprecipitated Beclin1. Band intensity of Beclin1 for IP/WCL in Figure S11B is shown. Data represent the mean and SD of three independent experiments. *p < 0.05 (paired Student’s *t*-test).
(TIF)

Movie S1 **FRAP analysis of EGFP-Rab39a on the phagosome.** This movie corresponds to Figure 1B. The phagosome surrounded by EGFP-Rab39a was photobleached at 4 sec, followed by monitoring the fluorescence recovery at 2 sec intervals.
(MOV)

Table S1 **Primer list for plasmid constructions.**
(XLS)

Table S2 **siRNA list for gene silencing.**
(XLS)

Table S3 **Primer list for real time-quantitative PCR.**
(XLS)

## References

[1] Schwartz SL, Cao C, Pylypenko O, Rak A, Wandinger-Ness A (2007) Rab GTPases at a glance. J Cell Sci 120: 3905-3910. doi:10.1242/jcs.015909. PubMed: 17989088. 17989088

[2] Stenmark H (2009) Rab GTPases as coordinators of vesicle traffic. Nat Rev Mol Cell Biol 10: 513-525. doi:10.1038/nrg2642. PubMed: 19603039. 19603039

[3] Chua CE, Gan BQ, Tang BL (2011) Involvement of members of the Rab family and related small GTPases in autophagosome formation and maturation. Cell Mol Life Sci 68: 3349-3358. doi:10.1007/s00018-011-0748-9. PubMed: 21687989. 21687989 PMC11114630

[4] Fairn GD, Grinstein S (2012) How nascent phagosomes mature to become phagolysosomes. Trends Immunol 33: 397-405. doi:10.1016/j.it.2012.03.003. PubMed: 22560866. 22560866

[5] Zerial M, McBride H (2001) Rab proteins as membrane organizers. Nat Rev Mol Cell Biol 2: 107-117. doi:10.1038/35052055. PubMed: 11252952. 11252952

[6] Hirota Y, Fujimoto K, Tanaka Y (2013) Rab GTPases in Autophagy. In: Bailly Y Autophagy - A Double-Edged Sword - Cell Survival or Death? InTech. pp. 47-63

[7] Seto S, Tsujimura K, Koide Y (2011) Rab GTPases regulating phagosome maturation are differentially recruited to mycobacterial phagosomes. Traffic 12: 407-420. doi:10.1111/j.1600-0854.2011.01165.x. PubMed: 21255211. 21255211

[8] Becker CE, Creagh EM, O'Neill LA (2009) Rab39a binds caspase-1 and is required for caspase-1-dependent interleukin-1beta secretion. J Biol Chem 284: 34531-34537. doi:10.1074/jbc.M109.046102. PubMed: 19833722. 19833722 PMC2787314

[9] Mori Y, Matsui T, Omote D, Fukuda M (2013) Small GTPase Rab39A interacts with UACA and regulates the retinoic acid-induced neurite morphology of Neuro2A cells. Biochem Biophys Res Commun 435: 113-119. doi:10.1016/j.bbrc.2013.04.051. PubMed: 23624502. 23624502

[10] Akira S, Takeda K, Kaisho T (2001) Toll-like receptors: critical proteins linking innate and acquired immunity. Nat Immunol 2: 675-680. doi:10.1038/90609. PubMed: 11477402. 11477402

[11] Beutler B (2004) Inferences, questions and possibilities in Toll-like receptor signalling. Nature 430: 257-263. doi:10.1038/nature02761. PubMed: 15241424. 15241424

[12] Hotchkiss RS, Karl IE (2003) The pathophysiology and treatment of sepsis. N Engl J Med 348: 138-150. doi:10.1056/NEJMra021333. PubMed: 12519925. 12519925

[13] Hotchkiss RS, Monneret G, Payen D (2013) Immunosuppression in sepsis: a novel understanding of the disorder and a new therapeutic approach. Lancet Infect Dis 13: 260-268. doi:10.1016/S1473-3099(13)70001-X. PubMed: 23427891. 23427891 PMC3798159

[14] Delgado MA, Elmaoued RA, Davis AS, Kyei G, Deretic V (2008) Toll-like receptors control autophagy. EMBO J 27: 1110-1121. doi:10.1038/emboj.2008.31. PubMed: 18337753. 18337753 PMC2323261

[15] Fujita K, Maeda D, Xiao Q, Srinivasula SM (2011) Nrf2-mediated induction of p62 controls Toll-like receptor-4-driven aggresome-like induced structure formation and autophagic degradation. Proc Natl Acad Sci U S A 108: 1427-1432. doi:10.1073/pnas.1014156108. PubMed: 21220332. 21220332 PMC3029726

[16] Into T, Inomata M, Takayama E, Takigawa T (2012) Autophagy in regulation of Toll-like receptor signaling. Cell Signal 24: 1150-1162. doi:10.1016/j.cellsig.2012.01.020. PubMed: 22333395. 22333395

[17] Sanjuan MA, Dillon CP, Tait SW, Moshiach S, Dorsey F et al. (2007) Toll-like receptor signalling in macrophages links the autophagy pathway to phagocytosis. Nature 450: 1253-1257. doi:10.1038/nature06421. PubMed: 18097414. 18097414

[18] Xu Y, Jagannath C, Liu XD, Sharafkhaneh A, Kolodziejska KE et al. (2007) Toll-like receptor 4 is a sensor for autophagy associated with innate immunity. Immunity 27: 135-144. doi:10.1016/j.immuni.2007.05.022. PubMed: 17658277. 17658277 PMC2680670

[19] Shi CS, Kehrl JH (2008) MyD88 and Trif target Beclin 1 to trigger autophagy in macrophages. J Biol Chem 283: 33175-33182. doi:10.1074/jbc.M804478200. PubMed: 18772134. 18772134 PMC2586260

[20] Saitoh T, Fujita N, Jang MH, Uematsu S, Yang BG et al. (2008) Loss of the autophagy protein Atg16L1 enhances endotoxin-induced IL-1beta production. Nature 456: 264-268. doi:10.1038/nature07383. PubMed: 18849965. 18849965

[21] Deretic V, Levine B (2009) Autophagy, immunity, and microbial adaptations. Cell Host Microbe 5: 527-549. doi:10.1016/j.chom.2009.05.016. PubMed: 19527881. 19527881 PMC2720763

[22] Itakura E, Kishi C, Inoue K, Mizushima N (2008) Beclin 1 forms two distinct phosphatidylinositol 3-kinase complexes with mammalian Atg14 and UVRAG. Mol Biol Cell 19: 5360-5372. doi:10.1091/mbc.E08-01-0080. PubMed: 18843052. 18843052 PMC2592660

[23] Seto S, Matsumoto S, Ohta I, Tsujimura K, Koide Y (2009) Dissection of Rab7 localization on Mycobacterium tuberculosis phagosome. Biochem Biophys Res Commun 387: 272-277. doi:10.1016/j.bbrc.2009.06.152. PubMed: 19580780. 19580780

[24] Seto S, Matsumoto S, Tsujimura K, Koide Y (2010) Differential recruitment of CD63 and Rab7-interacting-lysosomal-protein to phagosomes containing Mycobacterium tuberculosis in macrophages. Microbiol Immunol 54: 170-174. doi:10.1111/j.1348-0421.2010.00199.x. PubMed: 20236428. 20236428

[25] Sugaya K, Seto S, Tsujimura K, Koide Y (2011) Mobility of late endosomal and lysosomal markers on phagosomes analyzed by fluorescence recovery after photobleaching. Biochem Biophys Res Commun 410: 371-375. doi:10.1016/j.bbrc.2011.06.023. PubMed: 21683685. 21683685

[26] Seto S, Tsujimura K, Koide Y (2012) Coronin-1a inhibits autophagosome formation around Mycobacterium tuberculosis-containing phagosomes and assists mycobacterial survival in macrophages. Cell Microbiol 14: 710-727. doi:10.1111/j.1462-5822.2012.01754.x. PubMed: 22256790. 22256790

[27] Szeto J, Kaniuk NA, Canadien V, Nisman R, Mizushima N et al. (2006) ALIS are stress-induced protein storage compartments for substrates of the proteasome and autophagy. Autophagy 2: 189-199. PubMed: 16874109. Available online at: PubMed: 16874109 16874109 10.4161/auto.2731

[28] Behrends C, Sowa ME, Gygi SP, Harper JW (2010) Network organization of the human autophagy system. Nature 466: 68-76. doi:10.1038/nature09204. PubMed: 20562859. 20562859 PMC2901998

[29] Lindmo K, Stenmark H (2006) Regulation of membrane traffic by phosphoinositide 3-kinases. J Cell Sci 119: 605-614. doi:10.1242/jcs.02855. PubMed: 16467569. 16467569

[30] Seglen PO, Gordon PB (1982) 3-Methyladenine: specific inhibitor of autophagic/lysosomal protein degradation in isolated rat hepatocytes. Proc Natl Acad Sci U S A 79: 1889-1892. doi:10.1073/pnas.79.6.1889. PubMed: 6952238. 6952238 PMC346086

[31] Wu YT, Tan HL, Shui G, Bauvy C, Huang Q et al. (2010) Dual role of 3-methyladenine in modulation of autophagy via different temporal patterns of inhibition on class I and III phosphoinositide 3-kinase. J Biol Chem 285: 10850-10861. doi:10.1074/jbc.M109.080796. PubMed: 20123989. 20123989 PMC2856291

[32] Kang R, Zeh HJ, Lotze MT, Tang D (2011) The Beclin 1 network regulates autophagy and apoptosis. Cell Death Differ 18: 571-580. doi:10.1038/cdd.2010.191. PubMed: 21311563. 21311563 PMC3131912

[33] Giannandrea M, Bianchi V, Mignogna ML, Sirri A, Carrabino S et al. (2010) Mutations in the small GTPase gene RAB39B are responsible for X-linked mental retardation associated with autism, epilepsy, and macrocephaly. Am J Hum Genet 86: 185-195. doi:10.1016/j.ajhg.2010.01.011. PubMed: 20159109. 20159109 PMC2820185

[34] Takenaka M, Inoue H, Takeshima A, Kakura T, Hori T (2013) C. elegans Rassf homolog, rasf-1, is functionally associated with rab-39 Rab GTPase in oxidative stress response. Genes Cells 18: 203-210. doi:10.1111/gtc.12028. PubMed: 23294242. 23294242

[35] Wang C, Liu Z, Huang X (2012) Rab32 is important for autophagy and lipid storage in Drosophila. PLOS ONE 7: e32086. doi:10.1371/journal.pone.0032086. PubMed: 22348149. 22348149 PMC3279429

[36] Huynh KK, Eskelinen EL, Scott CC, Malevanets A, Saftig P et al. (2007) LAMP proteins are required for fusion of lysosomes with phagosomes. EMBO J 26: 313-324. doi:10.1038/sj.emboj.7601511. PubMed: 17245426. 17245426 PMC1783450

[37] Harris J (2013) Autophagy and IL-1 Family Cytokines. Front Immunol 4: 83 PubMed: 23577011. 23577011 10.3389/fimmu.2013.00083 PMC3617358

[38] Kawai T, Akira S (2007) Signaling to NF-kappaB by Toll-like receptors. Trends Mol Med 13: 460-469. doi:10.1016/j.molmed.2007.09.002. PubMed: 18029230. 18029230

[39] Kawai T, Akira S (2007) TLR signaling. Semin Immunol 19: 24-32. doi:10.1016/j.smim.2006.12.004. PubMed: 17275323. 17275323

[40] Copetti T, Bertoli C, Dalla E, Demarchi F, Schneider C (2009) p65/RelA modulates BECN1 transcription and autophagy. Mol Cell Biol 29: 2594-2608. doi:10.1128/MCB.01396-08. PubMed: 19289499. 19289499 PMC2682036

[41] Funderburk SF, Wang QJ, Yue Z (2010) The Beclin 1-VPS34 complex--at the crossroads of autophagy and beyond. Trends Cell Biol 20: 355-362. doi:10.1016/j.tcb.2010.03.002. PubMed: 20356743. 20356743 PMC3781210

[42] Matsunaga K, Saitoh T, Tabata K, Omori H, Satoh T et al. (2009) Two Beclin 1-binding proteins, Atg14L and Rubicon, reciprocally regulate autophagy at different stages. Nat Cell Biol 11: 385-396. doi:10.1038/ncb1846. PubMed: 19270696. 19270696

[43] Sun Q, Fan W, Chen K, Ding X, Chen S et al. (2008) Identification of Barkor as a mammalian autophagy-specific factor for Beclin 1 and class III phosphatidylinositol 3-kinase. Proc Natl Acad Sci U S A 105: 19211-19216. doi:10.1073/pnas.0810452105. PubMed: 19050071. 19050071 PMC2592986

[44] Zhong Y, Wang QJ, Li X, Yan Y, Backer JM et al. (2009) Distinct regulation of autophagic activity by Atg14L and Rubicon associated with Beclin 1-phosphatidylinositol-3-kinase complex. Nat Cell Biol 11: 468-476. doi:10.1038/ncb1854. PubMed: 19270693. 19270693 PMC2664389

[45] Stein MP, Feng Y, Cooper KL, Welford AM, Wandinger-Ness A (2003) Human VPS34 and p150 are Rab7 interacting partners. Traffic 4: 754-771. doi:10.1034/j.1600-0854.2003.00133.x. PubMed: 14617358. 14617358

[46] Sun Q, Westphal W, Wong KN, Tan I, Zhong Q (2010) Rubicon controls endosome maturation as a Rab7 effector. Proc Natl Acad Sci U S A 107: 19338-19343. doi:10.1073/pnas.1010554107. PubMed: 20974968. 20974968 PMC2984168

[47] Tabata K, Matsunaga K, Sakane A, Sasaki T, Noda T et al. (2010) Rubicon and PLEKHM1 negatively regulate the endocytic/autophagic pathway via a novel Rab7-binding domain. Mol Biol Cell 21: 4162-4172. doi:10.1091/mbc.E10-06-0495. PubMed: 20943950. 20943950 PMC2993745

[48] Nakamura N, Rabouille C, Watson R, Nilsson T, Hui N et al. (1995) Characterization of a cis-Golgi matrix protein, GM130. J Cell Biol 131: 1715-1726. doi:10.1083/jcb.131.6.1715. PubMed: 8557739. 8557739 PMC2120691

