# Supplementary figures and images for: Rab39a Interacts with Phosphatidylinositol 3-Kinase and Negatively Regulates Autophagy Induced by Lipopolysaccharide Stimulation in Macrophages
Source: PLoS One. 2013 Dec 13;8(12):e83324. doi: 10.1371/journal.pone.0083324 (PMC3862771; doi:10.1371/journal.pone.0083324)

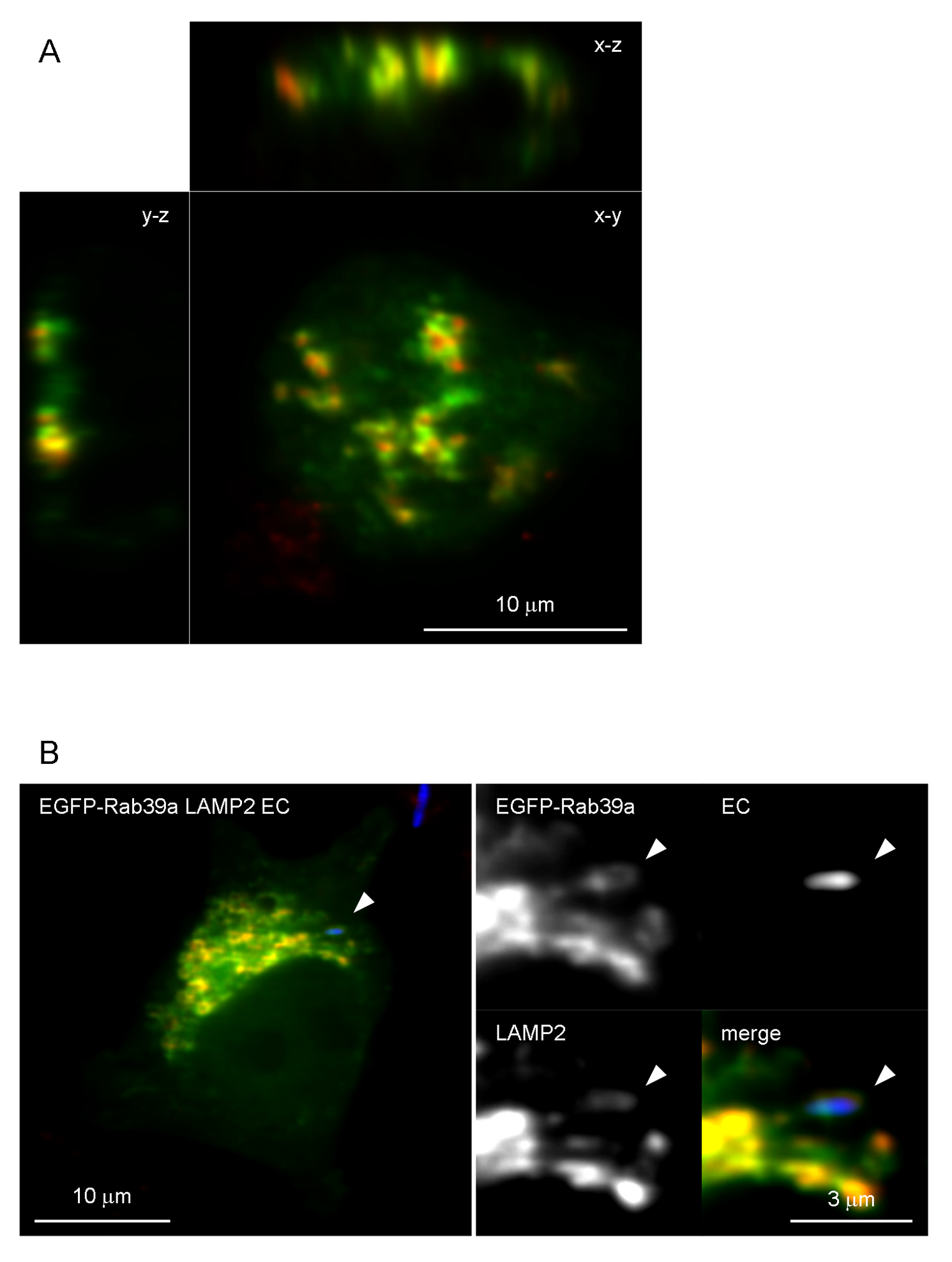

Supplement: Figure S1 — Rab39a localizes to lysosomes. (A) Projection of focal planes with y-z and x-z side views of Figure 1A is shown. (B) Localization of Rab39a in macrophages infected with E. coli. Raw264.7 macrophages expressing EGFP-Rab39a were infected with Alexa-405 labeled E. coli for 2 h and immunostained with anti-LAMP2 antibody. An arrowhead indicates a Rab39a-positive, LAMP2-positive E. coli-containing phagosome. (TIF) [file pone.0083324.s001.tif]

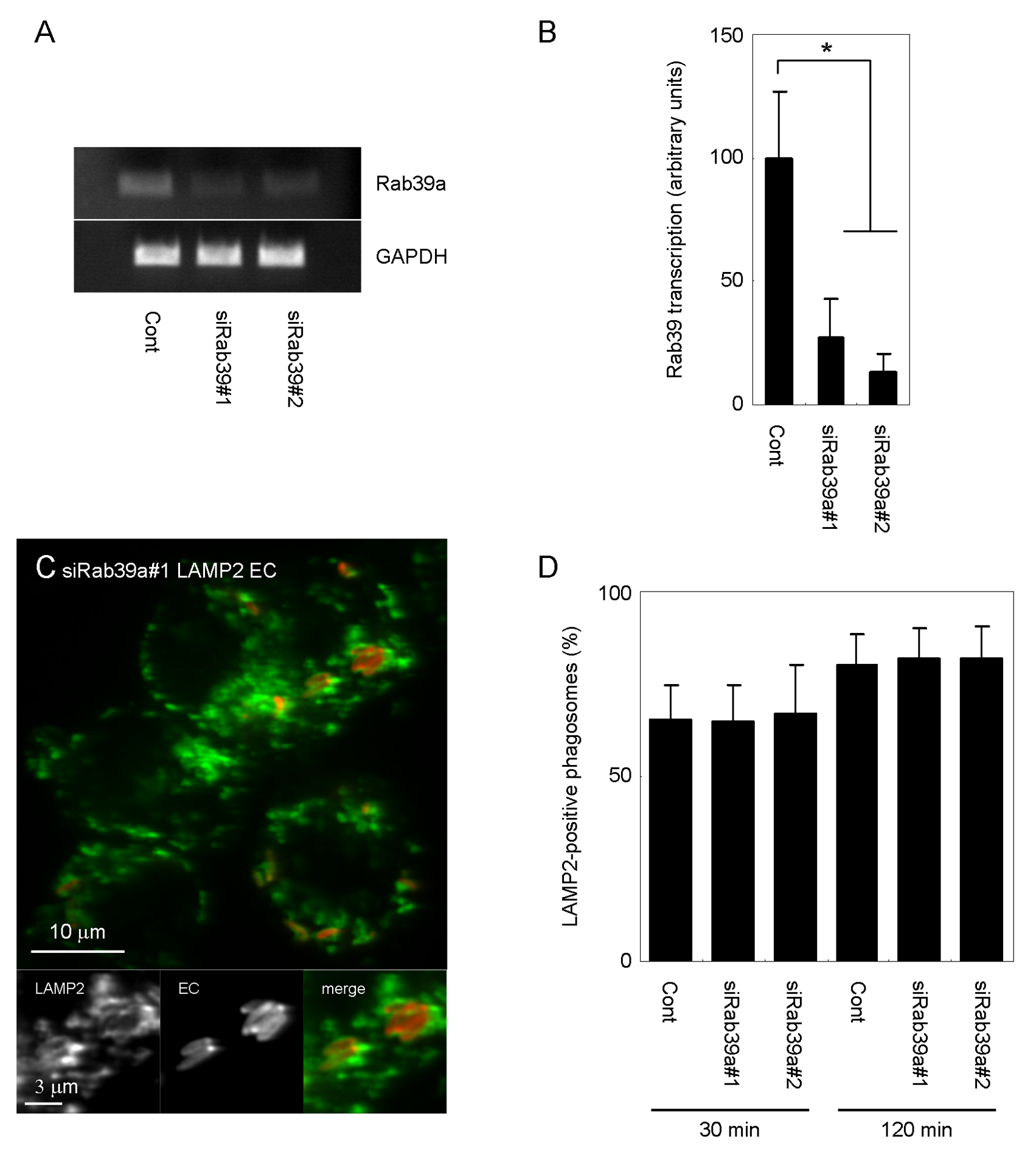

Supplement: Figure S2 — LAMP2 localization to phagosomes in Rab39a-KD macrophages. (A) RT-PCR analysis of Raw264.7 macrophages transfected with siRNA duplexes for Rab39a. (B) Quantitative analysis of Rab39 transcription. Rab39a mRNA level was assessed by RT-qPCR using GAPDH mRNA as a control. (C) Localization of LAMP2 to E. coli-containing phagosomes in Rab39a-KD macrophages. Raw264.7 macrophages transfected with siRNA for Rab39a were incubated with TexasRed-labeled E. coli for 2 h and were immunostained with anti-LAMP2 antibody. (D) The proportion of LAMP2-positive phagosomes in Rab39a-KD macrophages. Data represent the mean and SD of three independent experiments. *p < 0.05 (unpaired Student’s t-test). (TIF) [file pone.0083324.s002.tif]

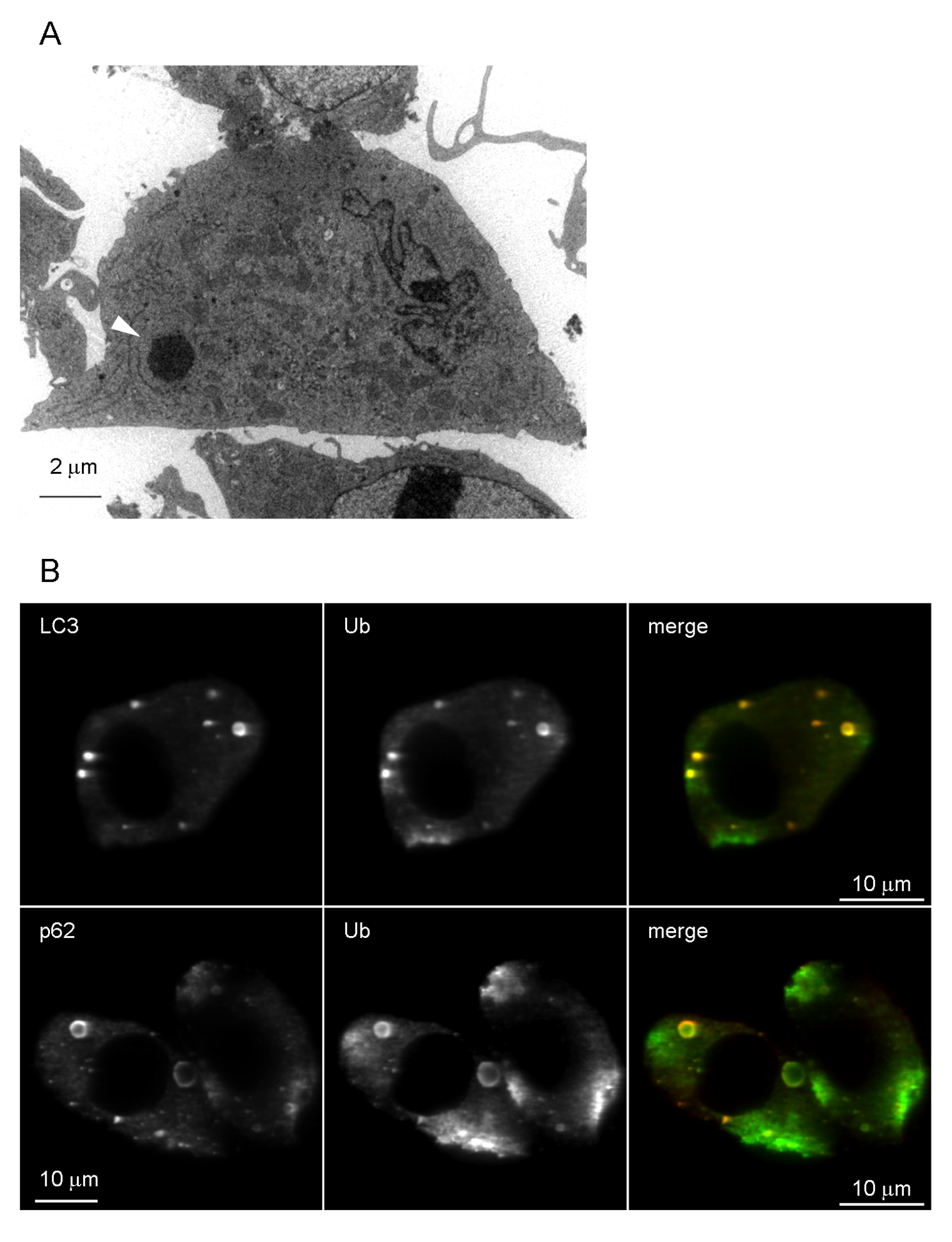

Supplement: Figure S4 — Characterization of LPS-induced autophagosome formation in Rab39a-KD macrophages. (A) Thin-section electron micrograph of Rab39a-KD macrophages. Raw264.7 macrophages transfected with control or Rab39a siRNA were treated with LPS at 10 ng/ml for 24 h and observed by thin-electron microscopy. An arrowhead indicates the electron dense-aggregation. (B) Co-localization of LC3 or p62 and ubiquitin in Rab39a-KD macrophages treated with LPS. Raw264.7 macrophages transfected with Rab39a siRNA were treated with LPS for 24 h and immunostained with anti-LC3 or anti-p62 and anti-ubiquitin antibodies. (TIF) [file pone.0083324.s004.tif]

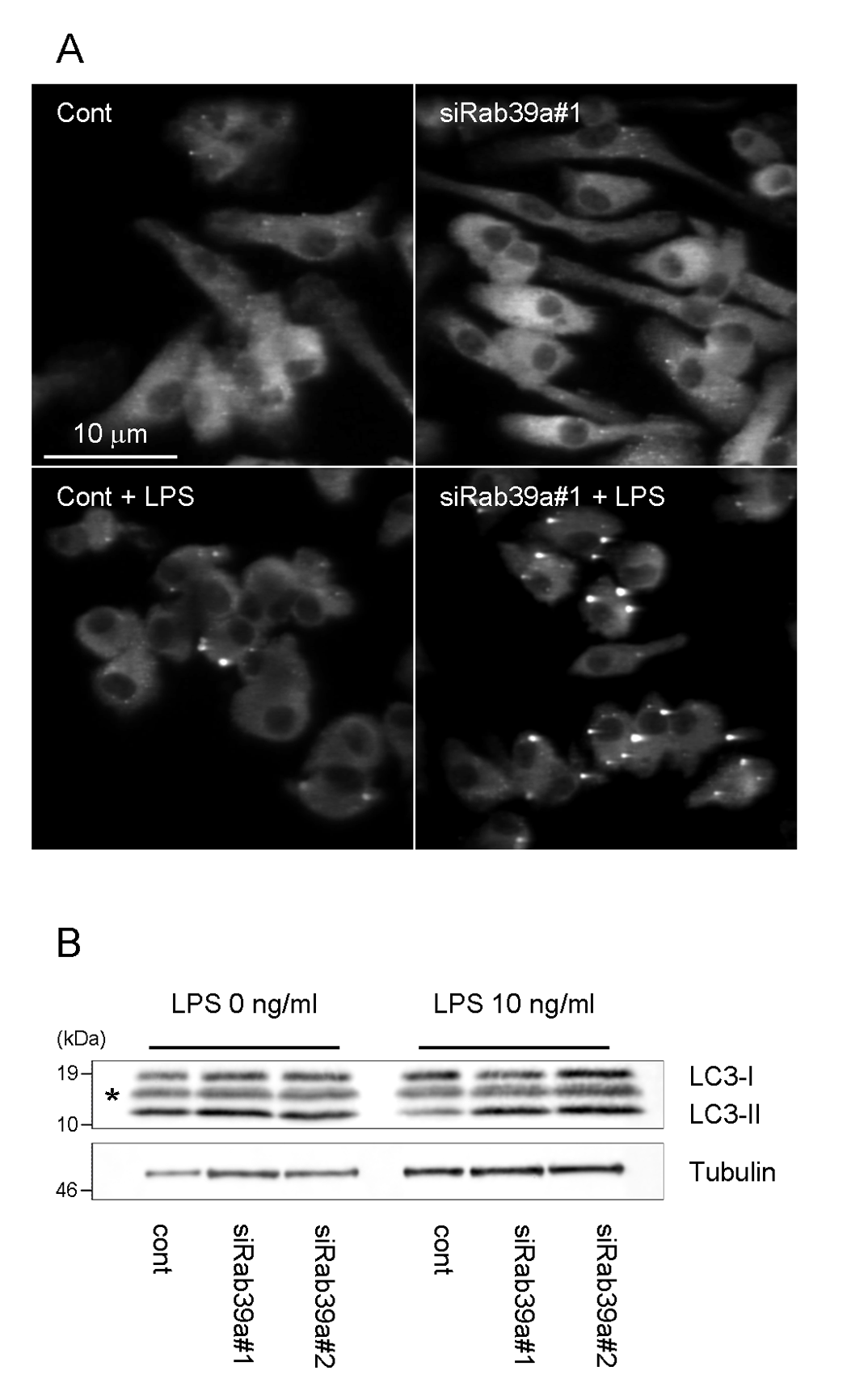

Supplement: Figure S5 — Augmentation of autophagy induced by LPS in Rab39a-KD BMM. (A) Immunofluorescence analysis of LC3 distribution. BMM transfected with control or Rab39a siRNA were treated with LPS for 24 h and immunostained with anti-LC3 antibody. (B) Immunoblot analysis of LC3 processing in BMM treated with LPS. Raw264.7 macrophages transfected with control or Rab39a siRNA were treated with LPS for 24 h and subjected to immunoblot analysis using indicated antibodies. *, non-specific band. (TIF) [file pone.0083324.s005.tif]

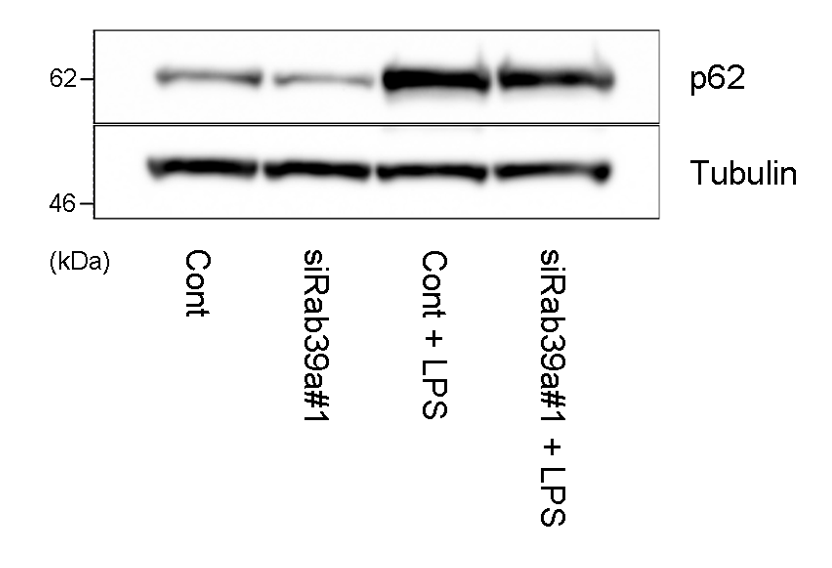

Supplement: Figure S7 — Expression level of p62 in Rab39a-KD macrophages. Raw264.7 macrophages transfected with control or Rab39a siRNA were treated with LPS for 24 h. Cell extracts were subjected to immunoblot analysis for anti-p62 antibody. (TIF) [file pone.0083324.s007.tif]

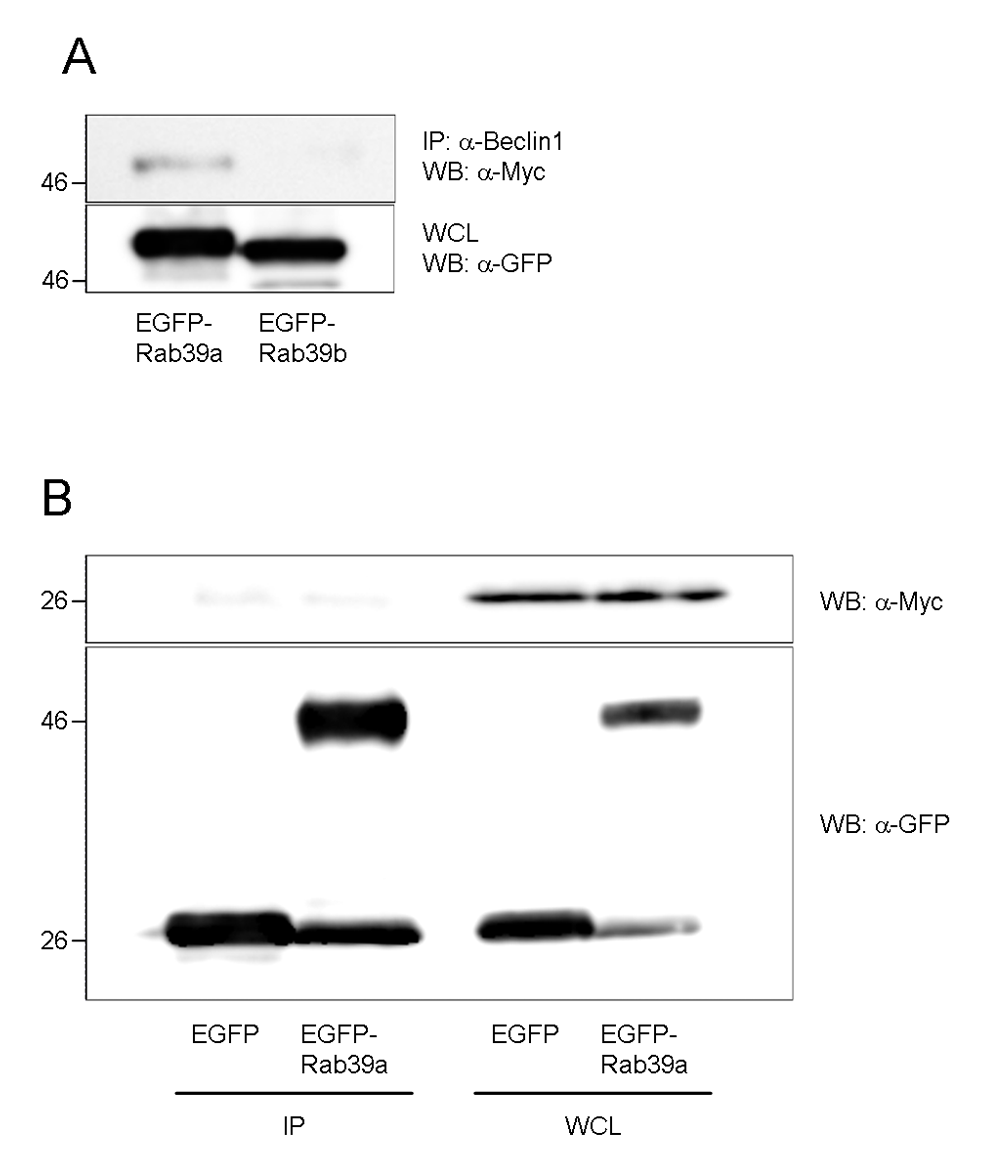

Supplement: Figure S8 — Interaction of Rab39a with Beclin1 or Bcl2. (A) HEK293T cells were transfected with plasmid for EGFP-Rab39a or EGFP-Rab39b. Whole cell lysates (WCL) were used for immunoprecipitation (IP) with anti-Beclin1 antibody, followed by immunoblot analysis (IB) with anti-GFP antibody. For detection of input, aliquots of 5 μg of WCL were used. (B) HEK293T cells were transfected with plasmids for Myc-Bcl2 and EGFP-Rab39a or EGFP-Rab39b. WCL were used for IP with anti-GFP antibody, followed by IB with anti-Myc antibody. For detection of input, aliquots of 5 μg of WCL were used. (TIF) [file pone.0083324.s008.tif]

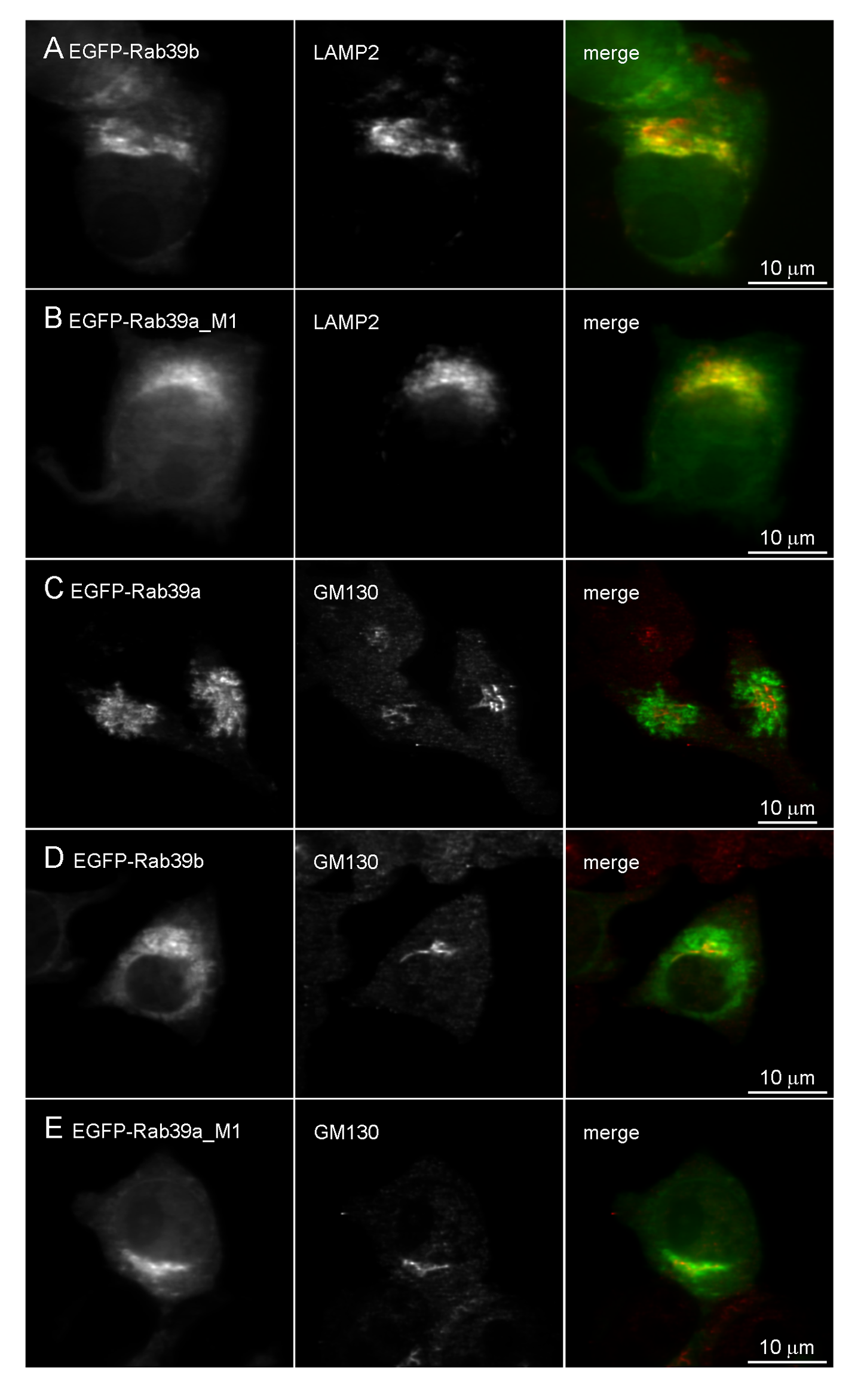

Supplement: Figure S9 — Subcellular localization of Rab39b and Rab39a_M1 in macrophages. Raw264.7 macrophages were transfected with the expression plasmid for EGFP-Rab39a, EGFP-Rab39b or EGFP-Rab39a_M1 and immunstained with anti-LAMP2 antibody (A, B) or anti-GM130 antibody (C, D, E). (TIF) [file pone.0083324.s009.tif]

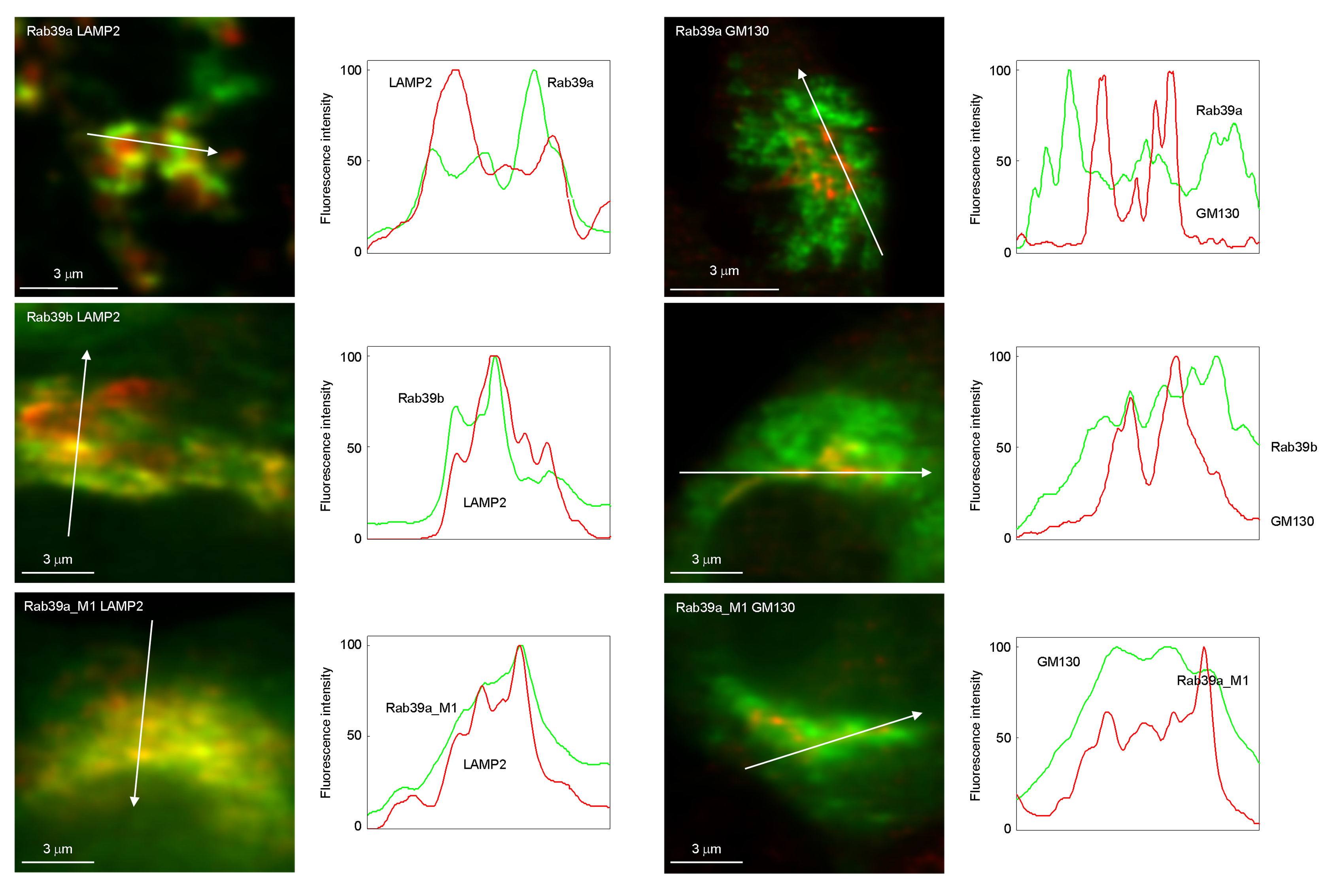

Supplement: Figure S10 — Enlarged images of Figures 1A and S5. Quantification for the fluorescence intensities across the arrows in right panels is also shown. (TIF) [file pone.0083324.s010.tif]

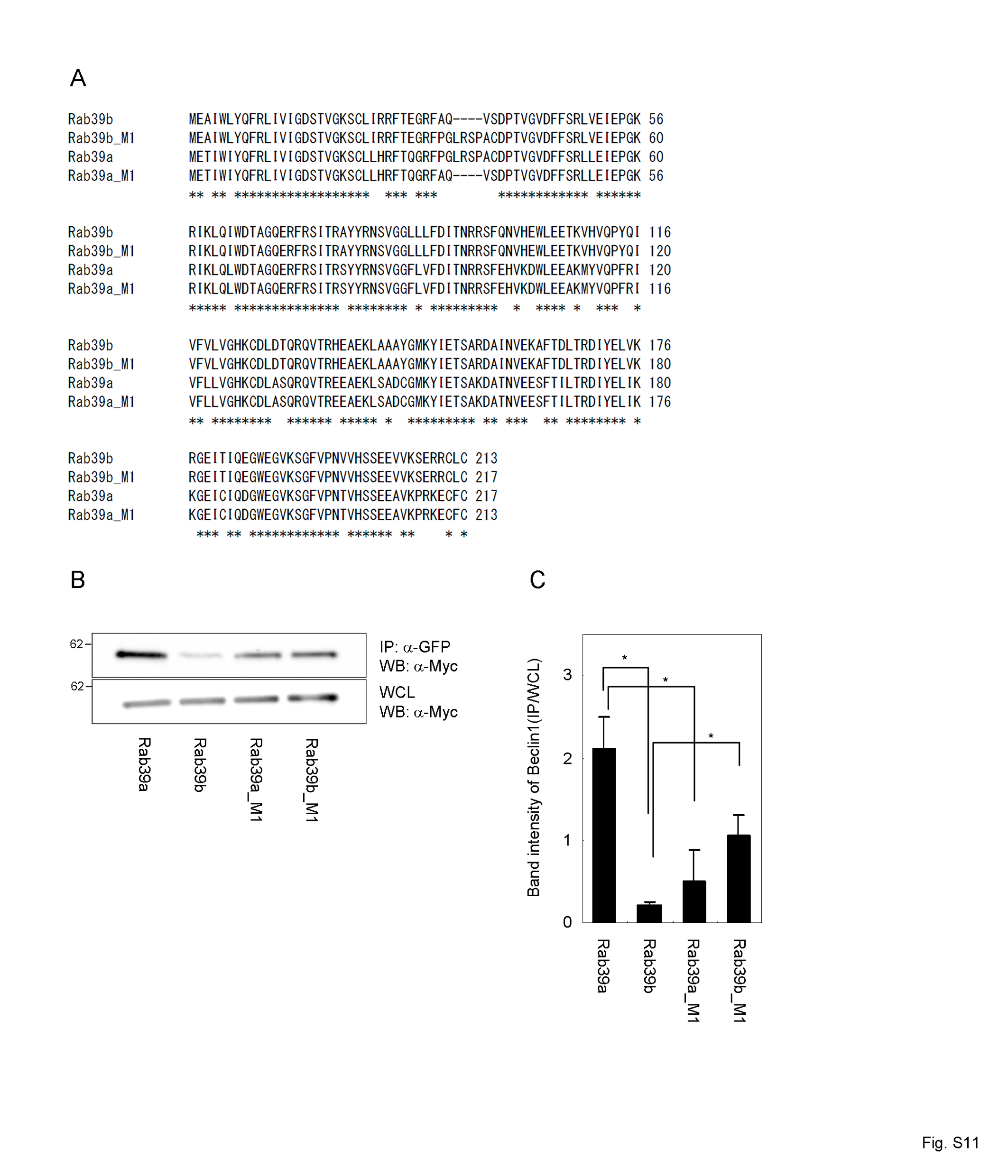

Supplement: Figure S11 — Amino acid residues from 34th to 41st of Rab39a is important for interaction of Rab39a with Beclin1. (A) ClustalW alignment of the amino acid sequences of Rab39a, Rab39b, Rab39a_M1 and Rab39b_M1 is shown. (B) HEK293T cells were transfected with plasmids for Myc-Beclin1 and EGFP-Rab39a, EGFP-Rab39b, EGFP-Rab39a_M1 or EGFP-Rab39b_M1. WCL were used for IP with anti-GFP antibody followed by IB with anti-Myc antibody. For detection of input, aliquots of 15 μg of WCL were used. (C) Quantification of band intensity for immunoprecipitated Beclin1. Band intensity of Beclin1 for IP/WCL in Figure S11B is shown. Data represent the mean and SD of three independent experiments. *p < 0.05 (paired Student’s t-test). (TIF) [file pone.0083324.s011.tif]
